# Palliative care and COVID-19: acknowledging past mistakes to forge a better future

**DOI:** 10.3389/fmed.2024.1390057

**Published:** 2024-07-25

**Authors:** Camila Rabelo Monteiro de Andrade, Fernanda Silva Trindade Luz, Neimy Ramos de Oliveira, Luciane Kopittke, Luiza Marinho Motta Santa Rosa, Angelica Gomides dos Reis Gomes, Frederico Bartolazzi, Saionara Cristina Francisco, Felicio Roberto da Costa, Alzira de Oliveira Jorge, Christiane Corrêa Rodrigues Cimini, Marcelo Carneiro, Karen Brasil Ruschel, Alexandre Vargas Schwarzbold, Daniela Ponce, Maria Angélica Pires Ferreira, Milton Henriques Guimarães Júnior, Daniel Vitório Silveira, Fernando Graça Aranha, Rafael Lima Rodrigues de Carvalho, Mariana Frizzo de Godoy, Lucas Macedo Pereira Viana, Vânia Naomi Hirakata, Maria Aparecida Camargos Bicalho, Milena Soriano Marcolino

**Affiliations:** ^1^Centro Universitário de Belo Horizonte, UniBH. Av. Professor Mário Werneck, Belo Horizonte, Brazil; ^2^Hospital Metropolitano Odilon Behrens. R. Formiga, Belo Horizonte, Brazil; ^3^Hospital Eduardo de Menezes. R. Dr. Cristiano Rezende, Belo Horizonte, Brazil; ^4^Hospital Nossa Senhora da Conceição. Av. Francisco Trein, Porto Alegre, Brazil; ^5^Hospital Cristo Redentor. R. Domingos Rubbo, Porto Alegre, Brazil; ^6^Faculdade Ciências Médicas de Minas Gerais. Al. Ezequiel Dias, Belo Horizonte, Brazil; ^7^Rede MaterDei de Saúde. Via Expressa, Betim, Brazil; ^8^Hospital Santo Antônio. R. Dr. Márcio de Carvalho Lopes, Curvelo, Brazil; ^9^Hospital Metropolitano Dr. Célio de Castro. R. Dona Luzia, Belo Horizonte, Brazil; ^10^Hospital Risoleta Tolentino Neves. R. das Gabirobas, Belo Horizonte, Brazil; ^11^Hospital Santa Rosália. R. Dr. Onofre, Teófilo Otoni, Brazil; ^12^Hospital Santa Cruz. Universidade de Santa Cruz do Sul. R. Fernando Abott, Santa Cruz do Sul, Brazil; ^13^Hospital Universitário Canoas. Av. Farroupilha, Canoas, Brazil; ^14^Hospital Mãe de Deus. R. José de Alencar, Porto Alegre, Brazil; ^15^Hospital Universitário de Santa Maria. Av. Roraima, Santa Maria, Brazil; ^16^Hospital das Clínicas da Faculdade de Medicina de Botucatu. Rod. Domingos Sartori, Botucatu, Brazil; ^17^Hospital de Clínicas de Porto Alegre. R. Ramiro Barcelos, Porto Alegre, Brazil; ^18^Hospital Márcio Cunha. Av. Eng. Kiyoshi Tsunawaki, Ipatinga, Brazil; ^19^Hospital Unimed-BH. Av. Contorno, Belo Horizonte, Brazil; ^20^Hospital SOS Cárdio. Rod. SC-401, Florianópolis, Brazil; ^21^Hospital Universitário Professor Edgard Santos. R. Augusto Viana, S/N, Salvador, Brazil; ^22^Escola de Enfermagem da Universidade Federal da Bahia. Basílio da Gama, Salvador, Bahia, Brazil; ^23^Hospital São Lucas da PUCRS. Av. Ipiranga, Porto Alegre, Brazil; ^24^Universidade Federal de Viçosa, Av. P H Rolfs, s/n - Campus Universitário, Viçosa, Brazil; ^25^Instituto Nacional de Ciência e Tecnologia Neurotec R. Av. Professor Alfredo Balena, Belo Horizonte, Brazil; ^26^Hospital João XXIII, Av. Prof. Alfredo Balena, Belo Horizonte, Brazil; ^27^Universidade Federal de Minas Gerais. Av. Professor Alfredo Balena, Belo Horizonte, Brazil; ^28^Fundação Hospitalar do Estado de Minas Gerais, FHEMIG. Al. Vereador Álvaro Celso, Belo Horizonte, Brazil; ^29^Department of Internal Medicine, Medical School & Telehealth Center, University Hospital, Universidade Federal de Minas Gerais. Av. Professor Alfredo Balena, Belo Horizonte, Brazil

**Keywords:** palliative care, COVID-19, clinical characteristics, frailty, hospitalization, outcomes assessment

## Abstract

**Context:**

COVID-19 induces complex distress across physical, psychological, and social realms and palliative care (PC) has the potential to mitigate this suffering significantly.

**Objectives:**

To describe the clinical characteristics and outcomes of COVID-19 patients with an indication of PC, compared to patients who had no indication, in different pandemic waves.

**Methods:**

This retrospective multicenter observational cohort included patients from 40 hospitals, admitted from March 2020 to August 2022. Patients who had an indication of palliative care (PC) described in their medical records were included in the palliative care group (PCG), while those who had no such indication in their medical records were allocated to the non-palliative care group (NPCG).

**Results:**

Out of 21,158 patients, only 6.7% had indication for PC registered in their medical records. The PCG was older, had a higher frequency of comorbidities, exhibited higher frailty, and had a higher prevalence of clinical complications and mortality (81.4% vs. 17.7%, *p* < 0.001), when compared to the NPCG. Regarding artificial life support, the PCG had a higher frequency of dialysis (20.4% vs. 10.1%, p < 0.001), invasive mechanical ventilation (48.2% vs. 26.0%, p < 0.001) and admission to the intensive care unit (53.6% vs. 35.4%, p < 0.001). These differences were consistent across all three waves.

**Conclusion:**

A low proportion of patients received PC. Patients in PCG were more fragile, had more clinical complications, and had a higher mortality. On the contrary to our expectations, they received more artificial life support in all three waves. Taken together, these findings suggest that decisions regarding PC indication were made too late, within a context of end-of-life and therapeutic failure.

## Introduction

The COVID-19 pandemic has had a significant impact on healthcare systems worldwide, with over 772 million cases and almost 7 million deaths globally (1). This unprecedented situation has led to several hospitalizations, stemming from either the direct impact of the infection or the exacerbation of pre-existing health conditions (2, 3).

Individuals afflicted by COVID-19, along with their families, endured multifaceted distress arising from severe physical symptoms related to the disease and its complications, and major psychological and social repercussions stemming from social isolation and other pandemic-related factors. In this context, palliative care (PC), a healthcare approach focused on alleviating suffering, is essential for providing better care (4).

Palliative care plays a crucial role in managing prevalent symptoms in COVID-19-infected patients, such as breathlessness and agitation, and involves empathetic communication with patients and families, whether in-person or through virtual platforms (5). Palliative care also aids clinicians in aligning the treatments provided with patients’ values and goals (6), and supports families in bereavement (7). Importantly, PC should not be contingent on the patient’s prognosis (8) because it is designed to integrate seamlessly with curative treatments (9).

Despite the clear benefits of PC, it was estimated that before COVID-19, less than 15% of people in need received it (10). The pandemic exacerbated the demand for health resources, including PC consultation, leading to a lower proportion of patients obtaining this care (11, 12), a situation particularly dire in low and middle-income countries, where health systems are generally less robust and have fewer resources compared to high-income countries (4).

During the pandemic, the demand for life support measures, such as invasive mechanical ventilation, dialysis, and ICU admissions, increased exponentially (13, 14). These interventions, while critical for some patients, may not align with the goals of care for those who are severely ill and have limited chances of recovery (15, 16). This discrepancy highlights the importance of early PC interventions to ensure that treatments are consistent with the patients’ wishes and overall health status (9, 16–19). By integrating PC early in the treatment course, healthcare providers can better manage symptoms, reduce unnecessary interventions, and improve the quality of life for patients and their families (7, 8, 11, 19). In this scenario, the pandemic highlighted the urgent need to optimize PC referrals and implement systematic protocols to ensure timely care (16, 20–22).

Studies assessing COVID-19 patients eligible for PC, considering their underlying diseases, clinical complications during hospitalization, and use of artificial life support, are scarce, particularly in developing countries outside Asia. Therefore, this study aims to describe the clinical characteristics and outcomes of COVID-19 in this population compared to patients who were not indicated for PC across the three different pandemic waves. The sample was collected in Brazil, a country severely hit by the COVID-19 pandemic (23).

Our initial hypothesis posited that patients in the PCG would be less likely to receive life support measures, given their lower likelihood of benefiting from them compared to patients in the non-palliative care group (NPCG), especially in a scenario of limited health resources.

## Methods

This is a substudy of the Brazilian COVID-19 Registry, a retrospective multicenter observational cohort that includes 40 Brazilian hospitals, funded by both public and private sources, located in 18 different cities, across six Brazilian states (Bahia, Minas Gerais, Pernambuco, Rio Grande do Sul, Santa Catarina, and São Paulo). The Brazilian COVID-19 Registry has been described in detail previously (24).

The study followed a predefined protocol (24). It adheres to the Strengthening the Reporting of Observational Studies in Epidemiology guidelines (25) and to the Helsinki Declaration. It has been approved by the Brazilian National Committee for Research Ethics (*Comissão Nacional de Ética em Pesquisa,* CONEP, CAAE: 30350820.5.1001.0008). The study was exempt from the individual free and informed consent term, considering its retrospective nature, the pandemic context, and the use of unidentified data.

### Study subjects

Consecutive patients aged 18 or older with laboratory-confirmed COVID-19 according to the World Health Organization guidance (26), admitted to one of the participating hospitals between March 2020 and August 2022, were included. Those who manifested COVID-19 symptoms during their hospital stay, who were transferred to other non-participant hospitals, or had missing value in the variable concerning PC indication were excluded. This study utilized a convenience sample, and *a priori* sample size calculation was not performed.

For the current analysis, we categorized patients into two distinct groups: (i) those with documented indications for PC in their medical records, referred to henceforth as the palliative care group (PCG); and (ii) those without any recorded indication for PC, denoted as the non-palliative care group (NPCG).

### Outcomes

The primary outcomes were the consumption of artificial life support measures, which included invasive mechanical ventilation (IMV) support and kidney replacement therapy (KRT). Secondary outcomes included admission to the intensive care unit (ICU), hospital length of stay, length of stay in the ICU, nosocomial infection, sepsis, thromboembolic events, and mortality.

### Measurement and data quality assessment

Demographic data, underlying comorbidities, clinical characteristics, laboratory and imaging exams, treatment, and outcomes were collected by previously trained personnel from the medical records (Supplementary material 1).

For frailty analysis, the Clinical Frailty Scale (CFS) was used as a reference (27), but the strata were regrouped into 6 groups: (i) fit (includes very fit, well, and managing well); (ii) vulnerable or mild frailty; (iii) moderate frailty; (iv) severe frailty (includes severely frail and very severely frail); (v) terminal illness (life expectancy <6 months); (vi) participants with no information described on medical reports. Patients were classified at hospital admission based on their pre-admission characteristics.

Collected data were included in specific forms on the Research Electronic Data Capture (REDCap) platform (28, 29), hosted in the Telehealth Center from the Universidade Federal de Minas Gerais (30). The data underwent periodic auditing to identify outliers and unexpected values. In instances where such discrepancies were detected, the data was returned to each local coordinator for double-checking, aiming to minimize errors and ensure data quality.

### Data analysis

For the descriptive analysis, demographic, clinical characteristics, and outcomes were represented by frequency distribution, using mean and standard deviations, as the sample size was large, while categorical data were described as absolute numbers and proportions.

Clinical characteristics and outcomes of PCG and NPCG were assessed during the whole period of data collection and stratified by COVID-19 waves. The first wave comprised the period from March 10th, 2020, to November 14th, 2020; the second wave was from November 15th, 2020, to December 25th, 2021, and the third wave was from December 26th, 2021, to March 8th, 2022 (31). The PCG was compared to NPCG using the t-test for continuous variables and Fisher’s exact test for categorical variables. In addition, patients’ characteristics were compared within each group, considering the three different waves, with the aim of identifying changes over the course of the pandemic. The significance level was set at a two-tailed *p*-value ≤ 0.05.

Since frailty assessments were conducted only during the second and third waves, participants from the first wave were excluded from the analysis of this variable. The same applies to vaccination data, which was available only from the second wave onward, as Brazil did not have access to vaccinations during the first wave.

Statistical analyses were conducted using IBM SPSS Statistics (IBM SPSS Statistics for Macintosh, Version 26.0 Armonk, NY: IBM Corp.RRID:SCR_019096).

## Results

Among 21,158 patients, 1,425 (6.7%) had a documented indication for PC in their medical records, with a higher frequency observed during the first wave (8%, 615/7,677), followed by the third (6.8%, 157/2,299), and the second wave (5.8%, 653/11,182). Patients in the PCG were older in both overall analysis (74 ± 14 vs. 58 ± 16 years) and stratified per wave analysis. There was a higher proportion of women (50.7% vs. 46.9%) compared to the NPCG in the overall analysis. This sex difference was observed in the first wave but not in the other waves (Table 1). Women were older in the overall analyses when compared to men, but this difference was not clinically relevant: 59 ± 17 vs. 58 ± 16 years (data not shown).

**Table 1:** Baseline demographics, underlying comorbidities and clinical characteristics at hospital presentation of COVID-19 patients with and without a palliative care indication registered in their medical records, including an overall comparison and a stratified analysis by COVID-19 waves.

	Overall comparison			Comparison through the waves						
Characteristics	NPCG (*N* = 19,733)	PCG (*N* = 1,425)	*p*-value^2^	First wave^1^		Second wave^1^		Third wave^1^		*p*-value^2^
				NPCG (*N* = 7,062)	PCG (*N* = 615)	NPCG (*N* = 10,529)	PCG (*N* = 653)	NPCG (*N* = 2,142)	PCG (*N* = 157)	
Age (years)	58 (±16)	74 (±14)	< 0.001	58 (±16)	75 (±13)	57 (±15)	72 (±14)	60 (±20)	77 (±14)	< 0.001
Women	9248^a^ (46.9%)	723^b^ (50.7%)	0.006	3194^a^ (45.2%)	334^b^ (54.3%)	4950^a^ (47.0%)	309^a^ (47.2%)	1104^a^ (51.5%)	80^a^ (51.0%)	< 0.001
Number of comorbidities			< 0.001							< 0.001
0	6392^a^ (32.4%)	234^b^ (16.4%)		2117^a^ (30.0%)	94^b^ (15.3%)	3635^a^ (34.5%)	113^b^ (17.3%)	640^a^ (29.9%)	27^b^ (17.2%)	
1	5965^a^ (30.2%)	405^a^ (28.4%)		2105^a^ (29.8%)	155^b^ (25.2%)	3266^a^ (31.0%)	202^a^ (30.9%)	594^a^ (27.7%)	48^a^ (30.6%)	
2	4673^a^ (23.7%)	448^b^ (31.4%)		1786^a^ (25.3%)	202^b^ (32.8%)	2391^a^ (22.7%)	204^b^ (31.5%)	496^a^ (23.2%)	40^a^ (25.5%)	
3	2051^a^ (10.4%)	224^b^ (15.7%)		775^a^ (11.0%)	104^b^ (16.9%)	995^a^ (9.5%)	91^b^ (13.9%)	281^a^ (13.1%)	29^a^ (18.5%)	
≥ 4	652^a^ (3.3%)	116^b^ (8.1%)		279^a^ (4.0%)	60^b^ (9.8%)	242^a^ (2.3%)	43^b^ (6.6%)	131^a^ (6.1%)	13^a^ (8.3%)	
Asthma	1130^a^ (5.7%)	57^b^ (4.0%)	0.006	435^a^ (6.2%)	27^a^ (4,4%)	575^a^ (5,5%)	25^a^ (3,8%)	120^a^ (5.6%)	5^a^ (3.2%)	0.077
AF or flutter	521^a^ (2.6%)	97^b^ (6.8%)	<0.001	218^a^ (3.1%)	53^b^ (8.6%)	203^a^ (1.9%)	34^b^ (5.2%)	100^a^ (4.7%)	10^a^ (6.4%)	<0.001
CAD	991^a^ (5.0%)	118^b^ (8.3%)	<0.001	395^a^ (5.6%)	58^b^ (9.4%)	423^a^ (4.0%)	48^b^ (7.3%)	173^a^ (8.1%)	12^a^ (7.6%)	<0.001
Cancer	877^a^ (4.4%)	222^b^ (15.6%)	<0.001	371^a^ (5.3%)	117^b^ (19.0%)	297^a^ (2.8%)	76^b^ (11.6%)	297^a^ (2.8%)	76^b^ (11.6%)	<0.001
Cirrhosis	116^a^ (0.6%)	28^b^ (2.0%)	<0.001	47^a^ (0.7%)	12^b^ (2.0%)	33^a^ (0.3%)	13^b^ (2.0%)	36^a^ (1.7%)	3^a^ (1.9%)	<0.001
CKD	967^a^ (4.9%)	154^b^ (10.8%)	<0.001	387^a^ (5.5%)	81^b^ (13.2%)	391^a^ (3.7%)	60^b^ (9.2%)	189^a^ (8.8%)	13^a^ (8.3%)	<0.001
COPD	1029^a^ (5.2%)	168^b^ (11.8%)	<0.001	410^a^ (5.8%)	82^b^ (13.3%)	398^a^ (3.8%)	68^b^ (10.4%)	221^a^ (10.3%)	18^a^ (11.5%)	<0.001
Dementia	375^a^ (1.9%)	211^b^ (14.8%)	<0.001	91^a^ (1.3%)	75^a^ (12.2%)	167^a^ (1.6%)	87^b^ (13.3%)	117^a^ (5.5%)	49^b^ (31.2%)	<0.001
Diabetes mellitus	5097^a^ (26.8%)	456^b^ (32.0%)	<0.001	2040^a^ (28.9%)	202^b^ (32.8%)	2655^a^ (25.2%)	203^b^ (31.0%)	602^a^ (28.1%)	51^a^ (35.2%)	0.038
Heart failure	1075^a^ (5.4%)	162^b^ (11.4%)	<0.001	427^a^ (6.0%)	87^b^ (14.1%)	424^a^ (4.0%)	55^b^ (8.4%)	224^a^ (10.5%)	20^a^ (12.7%)	<0.001
HIV infection	189^a^ (1.0%)	15^a^ (1.1%)	0.728	73^a^ (1.0%)	8^a^ (1.3%)	75^a^ (0.7%)	4^a^ (0.6%)	41^a^ (1.9%)	3^a^ (1.9%)	0.534
Hypertension	10233^a^ (51.9%)	905^b^ (64.4%)	<0.001	3788^a^ (53.6%)	396^b^ (64.4%)	5362^a^ (50.9%)	417^b^ (63.7%)	1083^a^ (50.6%)	92^a^ (58.6%)	<0.001
Stroke	631^a^ (3.2%)	176^b^ (12.3%)	<0.001	243^a^ (3.4%)	77^b^ (12.5%)	249^a^ (2.4%)	68^b^ (10.4%)	141^a^ (6.6%)	30^b^ (19.1%)	<0.001
Obesity	3483^a^ (17.7%)	143^b^ (10.0%)	<0.001	1267^a^ (17.9%)	46^b^ (7.5%)	2008^a^ (19.1%)	89^b^ (13.6%)	208^a^ (9.7%)	8^a^ (5.1%)	<0.001
Pulmonary fibrosis	71^a^ (0.4%)	17^b^ (1.2%)	<0.001	33^a^ (0.5%)	10^b^ (1.6%)	20^a^ (0.2%)	5^b^ (0.8%)	18^a^ (0.8%)	2^a^ (1.3%)	<0.001
COVID-19 vaccination										0.152
Yes	NA	NA	–	NA	NA	1,020 (9.6%)	82 (12.4%)	875 (38.6%)	56 (34.4%)	
Missing data	NA	NA	–	NA	NA	7,688 (72.3%)	467 (70.5%)	1,207 (52.5%)	100 (61.3%)	
Functional status^3^ fit	6450^a^ (84.4%)	196^b^ (36.3%)	<0.001	NA	NA	5792^a^ (87.8%)	183^b^ (43.8%)	658^a^ (59.6%)	13^b^ (11.9%)	<0.001
Frail or vulnerable	1249^a^ (16.2%)	336^b^ (62.2%)	<0.001	NA	NA	804^a^ (12.1%)	240^b^ (54.3%)	445^a^ (40.3%)	96^b^ (88.1%)	<0.001
Terminal illness	5^a^ (0.1%)	8^b^ (1.5%)	<0.001	NA	NA	4^a^ (0.1%)	8^b^ (1.9%)	1^a^ (0.1%)	0^a^ (0.0%)	0.157
Hospital presentation										
GCS < 15	1524^a^ (7.7%)	466^b^ (32.7%)	< 0.001	363^a^ (12.2%)	256^b^ (41.6%)	431^a^ (4.1%)	164^b^ (25.0%)	230^a^ (10.7%)	46^b^ (29.3%)	< 0.001
SF ratio	2588^a^ (13.0%)	276^b^ (19.3%)	< 0.001	952^a^ (13.8%)	131^b^ (22.1%)	1485^a^ (14.3%)	132^b^ (20.5%)	151^a^ (7.6%)	13^a^ (8.7%)	< 0.001
IMV	561^a^ (3.1%)	102^b^ (8.0%)	< 0.001	464^a^ (6.6%)	82^b^ (13.4%)	88^a^ (1.0%)	19^b^ (3.5%)	9^a^ (0.5%)	1^a^ (0.8%)	< 0.001
Systolic blood pressure										
> 90 mmHg	16665^a^ (96.0%)	1106^b^ (89.6%)	< 0.001	6284^a^ (93.5%)	495^b^ (84.2%)	8614^a^ (97.8%)	491^b^ (94.6%)	1767^a^ (96.9%)	120^a^ (94.5%)	2.283
< 90 mmHg	236^a^ (1.4%)	41^b^ (3.3%)	< 0.001	89^a^ (1.3%)	23^b^ (3.9%)	99^a^ (1.1%)	13^b^ (2.5%)	48^a^ (2.6%)	5^a^ (3.9%)	< 0.001
Inotrope use	450^a^ (2.6%)	87^b^ (7.1%)	< 0.001	346^a^ (5.1%)	70^b^ (11.9%)	95^a^ (1.1%)	15^b^ (2.9%)	9^a^ (0.05%)	2^a^ (1.6%)	0.194

Numbers are presented as n (%) or mean (standard deviation). AF, atrial fibrillation; CAD, coronary artery disease; CKD, chronic kidney disease; COPD, chronic obstructive pulmonary disease; GCS, Glasgow coma scale; HIV, human immunodeficiency virus; IMV, invasive mechanical ventilation; NA, not applicable, as vaccination was not available in Brazil at that time; SF ratio, Peripheral capillary oxygen saturation/fraction of inspired oxygen (SpO_2_/Fio_2_ Ratio). ^1^First wave: 10/03/2020 to 14/11/2020. Second wave: 15/11/2020 to 25/12/2021. Third wave: 26/12/2021 to 03/08/2022. ^2^If the *p*-value is significant, the superscript letters “a” and “b” inform in which comparison there is difference. If both groups have the same superscript letter, there is not statistically significance in that comparison. When each group has a different letter, there is a significant difference. ^3^Functional status: based on patients pre admission characteristics before falling ill with COVID-19.

Overall, the PCG exhibited a higher burden of comorbidities and a lower proportion of patients without comorbidities when compared to the NPCG (16.4% vs. 32.4%, *p* < 0.001, in the overall analysis). This was also observed in the stratified comparisons (Table 1). The overall prevalence of various comorbidities was higher in the PCG, except for asthma (4.0% vs. 5.7%, *p* = 0.006) and obesity (10.0% vs. 17.7%, *p* < 0.001), which were more frequent in the NPCG. While this difference persisted in the first and second waves, it became nonsignificant for several comorbidities in the third wave, such as cirrhosis, chronic kidney disease, chronic obstructive pulmonary disease, and heart failure. Cancer, stroke, and dementia were consistently more prevalent in the PCG across all three waves.

There was a high proportion of missing data concerning COVID-19 vaccination among the patient records (72.2% of cases during the second wave and 53.8% during the third wave). There was no significant difference in vaccination between groups in overall or stratified analyses (Table 1).

There was also a high proportion of missing data (38.9%) for frailty assessment during the period this data was collected (second and third waves). Among those, the PCG demonstrated greater frailty, with a higher proportion of vulnerable or frail patients, in both overall and stratified analyses. Conversely, the PCG had a lower proportion of fit individuals in overall analyses (36.3% vs. 84.4%, *p* < 0.001), as well as during the second (43.8% vs. 87.8%, *p* < 0.001) and third (11.9% vs. 59.6%, *p* < 0.001) waves (Table 1). Throughout the course of the pandemic, there was a significant reduction in the proportion of fit patients in both groups. In the NPCG, the proportion dropped from 87.8% in the second wave to 59.6% in the third, while in the PCG, it fell from 43.8 to 11.9% (data not shown). The proportion of patients with a life expectancy <6 months was significantly higher in the PCG (1.5% [8/538] vs. 0.1% [5/7,889], *p* < 0.001) in both overall and stratified analyses (Table 1).

Upon hospital presentation, dyspnea was the most prevalent symptom (Supplementary Table S1) in both groups and afflicted a significantly higher proportion of patients in the PCG during the first and third waves, but not in the second wave or in the overall analysis when compared to the NPCG. Impaired consciousness (Glasgow Coma Scale <15) at hospital presentation were more common in the PCG, when compared to the NPCG, in the overall (32.7% vs. 7.7%, *p* < 0.001), and in stratified analysis (Supplementary Table S1). Patients in PCG needed greater support upon hospital presentation, with higher use of IMV and vasoactive amines, in the overall comparison (8% vs. 3.1%, *p* < 0.001 and 7.1% vs. 2.6%, *p* < 0.001, respectively) and during the first and second waves. There was no significant difference between groups in the third wave (Table 1).

Regarding artificial life support during the entire period of hospitalization, when compared to the NPCG, the PCG received more KRT overall (20.4% vs. 10.1%, *p* < 0.001) and in the stratified analysis, with the following proportions: first wave (19.1% vs. 10.9%, *p* < 0.001), second wave (24.2% vs. 10.4%, *p* < 0.001), and third wave (9.6% vs. 5.7%, *p* < 0.001). The PCG group also required IMV more often: 48.2% vs. 26.0%, *p* < 0.001 in the overall analysis; 49.0% vs. 28.1%, *p* < 0.001 in the first wave; 51.6% vs. 27.3%, *p* < 0.001 in the second; and 31.4% vs. 12.9%, *p* < 0.001 in the third wave (Table 2). There was a significant reduction in the use of KRT and IMV in both groups during the third wave in comparison to the other waves (Supplementary Table S2).

**Table 2:** Outcomes of COVID-19 patients with palliative care indication in their medical records and those without the indication for palliative care, overall comparison and stratified analysis by COVID-19 waves.

	Overall comparison			Comparison through the waves						
Characteristics	NPCG (*N* = 19,733)	PCG (*N* = 1,425)	*p*-value^2^	First wave^1^		Second wave^1^		Third wave^1^		*p*-value^2^
				NPCG (*N* = 7,062)	PCG (*N* = 615)	NPCG (*N* = 10,529)	PCG (*N* = 653)	NPCG (*N* = 2,142)	PCG (*N* = 157)	
KRT	1983^a^ (10.1%)	291^b^ (20.4%)	<0.001	766^a^ (10.9%)	117^b^ (19.1%)	1097^a^ (10.4%)	158^b^ (24.2%)	121^a^ (5.7%)	15^b^ (9.6%)	<0.001
ICU admission	7,053 ^a^ (35.4%)	765^b^ (53.6%)	<0.001	2682^a^ (38.0%)	335^b^ (54.5%)	3779^a^ (35.9%)	364^b^ (55.8%)	527^a^ (24.7%)	65^b^ (41.4%)	<0.001
Time spent in the ICU (days)	9.0 (4.0–17.0)	12.0 (6.0–23.0)	<0.001	8.0 (4.0–16.0)	11.0 (6.0–20.0)	10.0 (5.0–17.0)	14.0 (7.0–24.0)	6.0 (2.0–14.0)	11.0 (4.0–18)	<0.001
ICU death^3^	2,865 (41.0%)	563 (73.5%)	<0.001	995 (14.1%)	248 (40.3%)	1691^a^ (16.1%)	271^b^ (41.3%)	184^a^ (8.6%)	43^b^ (27.4%)	<0.001
IMV	5084^a^ (26.0%)	685^b^ (48.2%)	<0.001	1942^a^ (28.1%)	299^b^ (49.0%)	2872^a^ (27.3%)	337^b^ (51.6%)	275^a^ (12.9%)	49^b^ (31.4%)	<0.001
Septic shock	2412^a^ (12.2%)	475^b^ (33.3%)	<0.001	887^a^ (12.6%)	195^b^ (31.7%)	1359^a^ (12.9%)	229^b^ (35.1%)	169^a^ (7.9%)	49^b^ (31.4%)	<0.001
Nosocomial infection	2546^a^ (12.9%)	396^b^ (27.8%)	<0.001	720^a^ (10.2%)	135^b^ (22.0%)	1572^a^ (14.9%)	212^b^ (32.5%)	257^a^ (12.0%)	48^b^ (30.8%)	<0.001
Acute heart failure	476^a^ (2.4%)	70^b^ (4.9%)	<0.001	175^a^ (2.5%)	36^b^ (5.9%)	225^a^ (2.1%)	25^b^ (3.8%)	76^a^ (3.6%)	8^a^ (5.1%)	<0.001
Thromboembolic events	1065^a^ (5.4%)	95^b^ (6.7%)	0.043	356^a^ (5.0%)	39^a^ (6.3%)	655^a^ (6.2%)	54^b^ (8.2%)	54^a^ (2.5%)	2^a^ (1.3%)	0.1
In-hospital stay (days)	8.0 (4.0–15.0)	13.0 (6.0–23.0)	<0.001	8.0 (4.0–14.0)	12.0 (6.0–20.0)	8.0 (5.0–15.2)	15.0 (7.0–26.0)	8.0 (4.0–17.0)	12.0 (6.0–21.0)	<0.001
In-hospital death	3518^a^ (17.7%)	1162^b^ (81.4%)	0.386	1183^a^ (16.8%)	486^b^ (79.0%)	2060^a^ (19.6%)	557^b^ (85.3%)	250^a^ (11.7%)	107^b^ (68.6%)	<0.001

^1^n (%); Median (IQR). ^2^Pearson’s Chi-squared test; Wilcoxon rank sum test; Fisher’s exact test. ICU, intensive care unit; IMV, invasive mechanical ventilation; KRT, kidney replacement therapy. ^1^First wave: 10/03/2020 to 14/11/2020. Second wave: 15/11/2020 to 25/12/2021. Third wave: 26/12/2021 to 03/08/2022. ^2^If the *p*-value is significant, the superscript letters “a” and “b” inform in which comparison there is difference. If both groups have the same superscript letter, there is not statistically significance in that comparison. When each group has a different letter, there is a significant difference. ^3^ICU death: deaths of patients who were admitted in the ICU.

Patients in the PCG were more frequently admitted to the ICU (53.6% vs. 35.4%, *p* < 0.001) when compared to the NPCG, and this difference was sustained across all three waves (Table 2). In the PCG, there was a significant reduction in the ICU beds used in the third wave (41.4%) when compared to the first (54.5%) and second (55.8%) waves (*p*=0.004). A similar trend was observed in the NPCG, with ICU admissions peaking in the first wave, showing intermediate values in the second wave, and reaching its lowest point in the third wave (Supplementary Table S2). Concerning clinical complications during hospitalization (Table 2), the PCG had a higher incidence of septic shock (33.3% vs. 12.2%, *p* < 0.001), nosocomial infection (27.8% vs. 12.9%, *p* < 0.001), and acute heart failure when compared to the NPCG (4.9% vs. 2.4%, *p* < 0.001) in overall analyses. In the stratified analysis, this difference remained significant for septic shock and nosocomial infection but not for acute heart failure in the third wave. In the analysis within the PCG, comparing the waves, there was no difference in the incidence of septic shock or acute heart failure. However, there was a higher incidence, within this group, of nosocomial infection in the second wave compared to the first wave (32.5% vs. 22%, *p* < 0.001; Supplementary Table S2). There was no significant difference in the incidence of thromboembolic events between groups in the overall analysis, but the PCG had a higher incidence of this event when compared to the NPCG in the second wave (Table 2).

Patients in the PCG had a longer hospital stay (13 [6–23] vs. 8 [4–15] days, *p* < 0.001) and spent more days in the ICU (12 [6–23] vs. 9 [4–17] days, *p* < 0.001) in overall and stratified analyses (Table 2), and there was no significant difference within the PCG in the wave comparison (Supplementary Table S2). This group also had a higher overall mortality rate (81.4% vs. 17.7%, *p* < 0.001), which was consistent in stratified analyses for the different waves (first 79% vs. 16.8%, *p* < 0.001; second 85.3% vs. 19.6%, p < 0.001; and third 68.6% vs. 11.7%, *p* < 0.001; Table 2). Comparing each group across the waves, mortality was significantly higher in the second wave when compared to the third wave for both groups (Supplementary Table S2). A higher proportion of patients who were admitted to the ICU died in this setting in the PCG, when compared to the NPCG (73.5% vs. 41%, *p* < 0.001) in the overall and stratified analyses (Table 2). And there was no significant difference in this outcome among the PCG across the waves (Supplementary Table S2).

## Discussion

Within this large cohort of COVID-19 hospitalized patients, we identified a notably small proportion of patients with indication for PC (less than 7%) based on medical records. Patients in the PCG were older, had more comorbidities, a higher frequency of frailty and clinical complications, and had a higher in-hospital mortality rate when compared to the NPCG, as anticipated. However, unexpectedly, this group received a greater extent of artificial life support measures, such as IMV and KRT, experienced an extended hospital stay, a heightened rate of ICU admissions, as well as a longer period in the ICU.

The shockingly low proportion of patients with an indication for PC in our cohort may be partially attributed to challenges associated with prognosticating an acute and largely unknown infectious disease (11). However, it could also stem from a lack of awareness regarding PC, its association with end-of-life care (32), and a sense of defeat among healthcare professionals primarily trained for curative purposes (33). The underutilization of PC services during the pandemic had been reported in other studies, but not to the extent that our findings suggest (11, 12, 18, 34–36). In a retrospective cohort conducted in the United States, it was revealed that less than 40% of critically ill patients admitted to the ICU with severe COVID-19 received a PC consultation (11). A Brazilian cohort study from two of the largest COVID-19 treatment centers in São Paulo (35) showed that 17% of COVID-19 patients had PC indication, which is significantly lower than previously reported by American data but is still nearly 2.5 times higher than our findings.

We have not collected data to ascertain how many patients with PC eligibility did not receive this care pathway. However, the substantial presence of patients grappling with conditions such as heart failure, active cancer, and dementia and afflicted with serious clinical complications such as septic shock in the NPCG leads us to surmise that a significant number of individuals may have been overlooked in terms of receiving compassionate care. This underutilization is likely influenced by several factors, including a limited understanding of this care pathway among the Brazilian general population, healthcare professionals, and policymakers (36) and the low availability of specialized teams, which are unequally distributed across the country and lacking integration (37, 38). In Brazil, the provision of PC is primarily concentrated in hospitals, but the majority of them do not have a PC team (37).

Based on a higher utilization of artificial life support and ICU beds, higher proportion of ICU deaths, longer hospital and longer ICU stay among patients in the PCG compared to the NPCG, we posit that PC was likely contemplated after the implementation of invasive measures or only after therapeutic failure and near death. These findings are in line with previous publications (11, 18, 34, 35) and may indicate that health professionals were more likely to recommend palliative care for patients with more severe COVID-19.

Data on frailty were collected from the second wave on, and it was noteworthy the high rate of missing data. We found a relevant prevalence of fit individuals in PCG, especially during the second wave, and very low rates of patients classified in the last strata of frailty, meaning a life expectancy of less than 6 months. The significant numbers of fit individuals in the PCG support our hypothesis that many patients had the indication for PC only after therapeutic failure. And the low proportion of patients classified as terminally ill, especially in an aging world where non-communicable diseases associated with frailty are leading causes of death (10), along with the high rate of missing data, may indicate a lack of professional preparedness in assessing frailty and valuing this parameter in the decision-making process.

The rapid and unpredictable progression of COVID-19 has led many patients to lose their ability to make decisions (8), making it even more challenging to construct a realistic care plan aligned with a patient’s preferences. We identified certain factors that could have impeded communication between patients and the healthcare team in the PCG right from hospital admission. While both groups exhibited high rates of dyspnea, the PCG had a higher prevalence of neurological manifestations and the use of mechanical ventilation. These factors may have hindered the identification of a patient’s pre-existing comorbidities, functional status, values, and wishes, which are essential elements for crafting an individualized care plan (39).

As predicted, patients in the PCG exhibited a heightened mortality rate. However, we observed a decline in this outcome during the third wave, coupled with reductions in ICU admissions, KRT usage, and Substitute for IMV administration. These positive trends were evident in both groups and may be linked to the widespread vaccination efforts and the prevalence of variants with lower lethality (40). We observed comparable vaccination rates in both groups, a finding that could have been influenced by the considerable amount of missing data. Vaccination status, akin to frailty, may have been undervalued, given the limited documentation of this information in the medical records, even in a setting of high comorbid rates.

It is important to emphasize that our study did not measure specialized delivery of PC, because this variable was not collected. Our study gathered data from medical records and included patients in PCG when any mention of this type of care was registered. This strategy aimed to analyze when the frontline healthcare professional considered PC. During the pandemic, the primary guiding document for PC in Brazil was the 41st resolution issued by the Brazilian Ministry of Health in 2018 (41). It provides guidelines for the organization of PC, in light of integrated continuous care, within the Brazilian Unified Health System *Sistema Único de Saúde* (SUS). This resolution specifies that care should be provided to individuals who are facing a life-threatening illness and, within a hospital setting, should aim to alleviate symptoms that cannot be managed through other means. Consequently, it implies that a significant proportion of COVID-19 patients requiring hospitalization should be included in this care provision, especially those admitted to an ICU.

Our analysis highlights a notably deficient identification of PC needs, which was considered mainly in the context of end-of-life and probably therapeutic failure and not as a way to provide comfort to frail patients with major comorbidities. PC is not about limiting life support, but about making the best, proportionate choice for each individual, considering technical parameters, patients` values, and goals of care. Therefore, its implementation could have mitigated considerable suffering and likely steered toward a more sustainable allocation of healthcare investments. We contend that our study offers a profound basis for reflection on the challenges of a recent past, aiming to better equip us for a future that effectively addresses human suffering arising from illness.

### Limitations

This study compared two groups based on PC indication from medical records data. However, as previously mentioned, the data collection did not assess the disparity between patients eligible for PC and those who ultimately received it, nor did it evaluate the quality of the care provided. Consequently, some patients may have received a palliative approach that was not documented in their medical records. Additionally, a significant percentage of patients lacked information about functionality, a crucial parameter for determining appropriate treatments.

### Strengths and perspectives

To the best of our acknowledgement, this is the largest study to investigate PC delivery in COVID-19 patients in Brazil and South American countries. Additionally, the inclusion of diverse centers from different regions enhances its applicability to a continental country marked by significant inequalities.

Future research endeavors should delve into the reasons behind defining PC, the availability of specialized care and education on PC, and the quality of care provided. This last dimension can be analyzed by considering data on symptom management, conflicts regarding goals of care, time elapsed between the indication of PC and death, and the time between patient admission to the hospital or ICU and the initiation of palliative care.

## Conclusion

Our cohort comprised a substantial number of patients from 40 public and private centers distributed across three macro-regions of Brazil. Only a small group of patients had an indication for PC based on medical reports. The PCG exhibited characteristics such as older age, increased frailty, and a higher prevalence of comorbidities, clinical complications, and mortality compared to the NPCG. They also had higher usage of artificial life support, which contradicted our initial predictions, considering that patients were frailer and had a higher number of comorbidities, making them seemingly less likely to benefit from such measures. Additionally, we observed a low emphasis on functional assessment, leading us to speculate that this parameter was given minimal consideration in the decision-making process.

## Data availability statement

The original contributions presented in the study are included in the article/Supplementary material, further inquiries can be directed to the corresponding author.

## Ethics statement

This study was approved by the Brazilian National Committee for Research Ethics (*Comissão Nacional de Ética em Pesquisa*, CONEP, CAAE: 30350820.5.1001.0008). It was exempt from the individual free and informed consent term, considering its retrospective nature, the pandemic context, and the use of unidentified data.

## Author contributions

CA: Conceptualization, Data curation, Formal analysis, Investigation, Methodology, Project administration, Supervision, Writing – original draft, Writing – review & editing. FL: Conceptualization, Data curation, Formal analysis, Investigation, Methodology, Project administration, Supervision, Writing – original draft, Writing – review & editing. NO: Conceptualization, Data curation, Formal analysis, Investigation, Methodology, Project administration, Supervision, Writing – original draft, Writing – review & editing. LK: Conceptualization, Data curation, Formal analysis, Investigation, Methodology, Project administration, Supervision, Writing – original draft, Writing – review & editing. LSR: Conceptualization, Data curation, Methodology, Writing – original draft, Writing – review & editing. AG: Data curation, Writing – review & editing. FB: Data curation, Writing – review & editing. SF: Data curation, Writing – review & editing. FC: Data curation, Writing – review & editing. AJ: Data curation, Writing – review & editing. CC: Data curation, Writing – review & editing. MC: Data curation, Writing – review & editing. KR: Data curation, Writing – review & editing. AS: Data curation, Writing – review & editing. DP: Data curation, Writing – review & editing. MF: Data curation, Writing – review & editing. MGJ: Data curation, Writing – review & editing. DS: Data curation, Writing – review & editing. FA: Data curation, Writing – review & editing. RC: Data curation, Writing – review & editing. MG: Data curation, Writing – review & editing. MB: Data curation, Writing – review & editing, Conceptualization, Investigation, Methodology, Project administration, Supervision, Writing – original draft. MM: Conceptualization, Data curation, Formal analysis, Funding acquisition, Investigation, Methodology, Project administration, Resources, Software, Supervision, Validation, Visualization, Writing – original draft, Writing – review & editing. LV: Data curation, Writing – review & editing. VH: Formal analysis, Writing – review & editing.
