# Supplementary material for: Palliative care and COVID-19: acknowledging past mistakes to forge a better future
Source: Front Med (Lausanne). 2024 Jul 25;11:1390057. doi: 10.3389/fmed.2024.1390057 (PMC11306131; doi:10.3389/fmed.2024.1390057)
Supplement: Supplementary file 2 [file Data_Sheet_2.pdf]

Supplementary Table S2

PALLIATIVE CARE AND COVID-19: ACKNOWLEDGING PAST MISTAKES TO FORGE A BETTER FUTURE

AUTHORS

Camila Rabelo Monteiro de Andrade, MD [1] (Andrade, CRM; camilapep@gmail.com; 0009-0005-6869-1076)

Fernanda Silva Trindade Luz, MD [2] (Luz, FST; fernandatrindade.med@gmail.com; 0000-0003-4309-9998)

Neimy Ramos de Oliveira, MD [3] (Oliveira, NR; neimyramos@gmail.com); 0000-0001-5408-9459)

Luciane Kopittke, Pharm, MSc, PhD [4,5] (Kopittke L; lucianekopittke@gmail.com; 0000-0002-6606-7756)

Luiza Marinho Motta Santa Rosa [6] (Rosa, LMMS; luiza.motta26@gmail.com; 0000-0002-4741-4871)

Angelica Gomides dos Reis Gomes, MD, MSc [7] (Gomes, AGR; angelicagrgomes@gmail.com; 0000-0002-4568-0738)

Frederico Bartolazzi, MD, MSc [8] (Bartolazzi, F; fredlazzi@hotmail.com; 0000-0002-9696-4685)

Saionara Cristina Francisco, BSc, MSc [9] (Francisco, SC; saionaracf@gmail.com; 0000-0002-9655-6294)

Felicio Roberto da Costa, MD [2] (Costa, FR; felicio_roberto@hotmail.com; 0000-0001-9923-236X)

Alzira de Oliveira Jorge, MD, MSc, PhD [10] (Jorge, AO; alzira.o.jorge@gmail.com; 0000-0003-1366-1732)

Christiane Corrêa Rodrigues Cimini, MD, MSc, PhD [11] (Cimini, CCR; christiane.cimini@gmail.com; 0000-0002-1973-1343)

Marcelo Carneiro, MD, MSc, PhD [12] (Carneiro, M; marceloc@unisc.br; 0000-0003-3603-1987)

Karen Brasil Ruschel, RN, MSc, PhD [13,14] (Ruschel, KB; karenbruschel@gmail.com; 0000-0002-0812-920X)

Alexandre Vargas Schwarzbold, MD, MSc, PhD [15] (Schwarzbold, AV; alexvspoa@gmail.com; 0000-0002-5535-6288)

Daniela Ponce, MD, MSc, PhD [16] (Ponce, D; daniela.ponce@unesp.br; 0000-0002-6178-6938)

Maria Angélica Pires Ferreira, MD, MSc, PhD [17] (Ferreira, MAPF; mpiferreira@hcpa.edu.br; 0000-0003-0961-524X)

Milton Henriques Guimarães Júnior, MD, MSc [18] (Guimarães-Júnior, MH; miltonhenriques@yahoo.com.br; 0000-0002-2127-8015)

Daniel Vitório Silveira, MD, MSc [19] (Silveira, DV; danielvez@gmail.com; 0000-0002-7381-1651)

Fernando Graça Aranha, MD, MSc [20] (Aranha, FG; fgaranha2012@gmail.com; 0000-0001-9173-8892)

Rafael Lima Rodrigues de Carvalho, BSc, MSc, PhD [21,22] (Carvalho, RLR; rafaelsjdr@hotmail.com; 0000-0003-3576-3748)

Mariana Frizzo de Godoy, MD [23] (Godoy, MF; mfdegodoy@gmail.com; 0000-0002-6631-8826)

Lucas Macedo Pereira Viana[24] (Viana, LMP; lmacedopv@gmail.com; 0000-0002-8457-0531)

Vânia Naomi Hirakata, BSc, MSc[4] (Hirakata, VN; vhirakata@hcpa.edu.br; 0000-0003-4645-2080)

Maria Aparecida Camargos Bicalho, MD, MSc, PhD [25,26,27,28] (Bicalho, MAC; macbicalho@gmail.com; 0000-0001-6298-9377)

Milena Soriano Marcolino MD, MSc, PhD [27,29] (Marcolino MS; milenamarc@gmail.com; 0000-0003-4278-3771)

INSTITUTIONS

[1] Centro Universitário de Belo Horizonte, UniBH. Av. Professor Mário Werneck, 1685, Belo Horizonte, Brazil.

[2] Hospital Metropolitano Odilon Behrens. R. Formiga, 50. Belo Horizonte, Brazil.

[3] Hospital Eduardo de Menezes. R. Dr. Cristiano Rezende, 2213, Belo Horizonte, Brazil.

[4] Hospital Nossa Senhora da Conceição. Av. Francisco Trein, 326, Porto Alegre, Brazil.

[5] Hospital Cristo Redentor. R. Domingos Rubbo, 20, Porto Alegre, Brazil.

[6] Faculdade Ciências Médicas de Minas Gerais. Al. Ezequiel Dias, 275, Belo Horizonte, Brazil.

[7] Rede MaterDei de Saúde. Via Expressa, 15500, Betim, Brazil.

[8] Hospital Santo Antônio. R. Dr. Márcio de Carvalho Lopes, 501, Curvelo, Brazil.

[9] Hospital Metropolitano Dr. Célio de Castro. R. Dona Luzia, 311, Belo Horizonte, Brazil.

[10] Hospital Risoleta Tolentino Neves. R. das Gabirobas, 1, Belo Horizonte, Brazil.

[11] Hospital Santa Rosália. R. Dr. Onofre, 575, Teófilo Otoni, Brazil.

[12] Hospital Santa Cruz. Universidade de Santa Cruz do Sul. R. Fernando Abott, 174, Santa Cruz do Sul, Brazil.

[13] Hospital Universitário Canoas. Av. Farroupilha, 8001, Canoas, Brazil.

[14] Hospital Mãe de Deus. R. José de Alencar, 286, Porto Alegre, Brazil.

[15] Hospital Universitário de Santa Maria. Av. Roraima, 1000, Santa Maria, Brazil.

[16] Hospital das Clínicas da Faculdade de Medicina de Botucatu. Rod. Domingos Sartori, 21500, Botucatu, Brazil.

[17] Hospital de Clínicas de Porto Alegre. R. Ramiro Barcelos, 2350, Porto Alegre, Brazil.

[18] Hospital Márcio Cunha. Av. Eng. Kiyoshi Tsunawaki, 41, Ipatinga, Brazil.

[19] Hospital Unimed-BH. Av. Contorno, 3097, Belo Horizonte, Brazil.

[20] Hospital SOS Cárdio. Rod. SC-401, 121, Florianópolis, Brazil.

[21] Hospital Universitário Professor Edgard Santos. R. Augusto Viana, S/N, Salvador, Brazil.

[22] Escola de Enfermagem da Universidade Federal da Bahia. Basílio da Gama, 241. Salvador, Bahia, Brazil.

[23] Hospital São Lucas da PUCRS. Av. Ipiranga 6690, Porto Alegre, Brazil.

[24] Instituto Nacional de Ciência e Tecnologia Neurotec R. Av. Professor Alfredo Balena, 110, room 114, Belo Horizonte, Brazil.

[25] Hospital João XXIII, Av. Prof. Alfredo Balena, Belo Horizonte, Brazil.

[26] Universidade Federal de Minas Gerais. Av. Professor Alfredo Balena, 110, Belo Horizonte, Brazil.

[27] Fundação Hospitalar do Estado de Minas Gerais, FHEMIG. Al. Vereador Álvaro Celso, 100, Belo Horizonte, Brazil.

[28] Department of Internal Medicine, Medical School & Telehealth Center, University Hospital, Universidade Federal de Minas Gerais. Av. Professor Alfredo Balena, 110, Belo Horizonte, Brazil.

CORRESPONDING AUTHOR

Camila Rabelo Monteiro de Andrade

Centro Universitário de Belo Horizonte, UniBH.

Av. Professor Mário Werneck, 1685, Belo Horizonte, Brazil.

CEP 30455-610

E-mail:camilapep@gmail.co

Table S2. Comparative outcomes of COVID-19 patients across the waves within the two groups: palliative care group and non-palliative care group

Characteristics	**PCG** (N=1,427)			p-value[2]	**NPCG** (N=19,776)			p-value[2]
	**First wave[1]** (N=615)	**Second wave[1]** (N=655)	**Third wave[1]** (N=157)		**First wave[1]** (N=7,062)	**Second wave[1]** (N=10,528)	**Third wave[1]** (N=2,142)	
KRT	117[a] (19.1%)	158[a] (24.1%)	16[b] (10.3%)	<0.001	767[a] (10.9%)	1099[a] (10.4%)	122[b] (5.6%)	<0.001
ICU admission	335[a] (54.5%)	366[a] (55.8%)	68[b] (43.3%)	0.012	2692[a] (38.1%)	3812[b] (36.2%)	549[c] (25.6%)	<0.001
Time spent in the ICU (days)	11.0 (6.0 – 20.0)	14.0 (7.0 – 24.0)	11.0 (4.0 – 18.0)	0.03	8.0 (4.0 – 16.0)	10.0 (5.0 – 17.0)	6.0 (2.0 – 14.0)	0.03
In-hospital ICU death	248[a] (40.3%)	271[a] (41.3%)	43[a] (27.4%)	<0.001	995 [a] (14.1%)	1691 [b] (16.1%)	184 [a] (8.6%)	<0.001
IMV	299[a] (49.0%)	339[b] (51.4%)	50[b] (31.6%)	<0.001	1945[a] (28.0%)	2887[a] (27.2%)	284[b] (12.6%)	<0.001
Septic shock	195[a] (31.7%)	232[a] (35.2%)	51[a] (32.3%)	0.397	887[a] (12.5%)	1367[a] (12.9%)	173[b] (7.7%)	<0.001
Nosocomial infection	135[a] (22.0%)	215[b] (32.6%)	49[a,b] (31.0%)	<0.001	719[a] (10.1%)	1581[b] (14.9%)	261[a] (11.6%)	<0.001
Acute heart failure	36[a] (5.9%)	25[a] (3.8%)	9[a] (5.7%)	0.207	175[a] (2.5%)	225[a] (2.1%)	78[b] (3.5%)	0.001
Thromboembolic events	39[a] (6.3%)	54[a] (8.2%)	2[b] (1.3%)	0.007	355[a] (5.0%)	656[b] (6.2%)	54[c] (2.4%)	<0.001

In-hospital stay (days)	12.0 (6.0 – 20.0)	15.0 (7.0 – 26.0)	12.0 (6.0 – 21.0)	<0.001	8.0 (4.0 – 14.0)	8.0 (5.0 – 15.2)	8.0 (4.0 – 17.0)	<0.001
In-hospital death	486[a] (79.0%)	561[b] (85.6%)	108[c] (68.8%)	<0.001	1181[a] (16.7%)	2067[b] (19.6%)	254[c] (11.9%)	<0.001

[1]n (%); Median (IQR) [2]Pearson's Chi-squared test; Wilcoxon rank sum test; Fisher's exact test.

ICU: intensive care unit; IMV: invasive mechanical ventilation; KRT: kidney replacement therapy.

[1] First wave: 10/03/2020 to 14/11/2020. Second wave: 15/11/2020 to 25/12/2021. Third wave: 26/12/2021 to 03/08/2022.

[2] If the p-value is significant, the superscript letters "a" and "b" inform in which comparison there is difference. If both groups have the same superscript letter, there is not statistically significance in that comparison. When each group has a different letter, there is a significant difference. If a group has "a" and "b" it means a value between the other two groups with no difference with either one.
