# Supplementary material for: Palliative care and COVID-19: acknowledging past mistakes to forge a better future
Source: Front Med (Lausanne). 2024 Jul 25;11:1390057. doi: 10.3389/fmed.2024.1390057 (PMC11306131; doi:10.3389/fmed.2024.1390057)
Supplement: Supplementary file 3 [file Data_Sheet_3.pdf]

frontiers

Supplementary Material 1

PALLIATIVE CARE AND COVID-19: ACKNOWLEDGING PAST MISTAKES TO FORGE A BETTER FUTURE

AUTHORS

Camila Rabelo Monteiro de Andrade, MD [1] (Andrade, CRM; camilapep@gmail.com; 0009-0005-6869-1076)

Fernanda Silva Trindade Luz, MD [2] (Luz, FST; fernandatrindade.med@gmail.com; 0000-0003-4309-9998)

Neimy Ramos de Oliveira, MD [3] (Oliveira, NR; neimyramos@gmail.com); 0000-0001-5408-9459)

Luciane Kopittke, Pharm, MSc, PhD [4,5] (Kopittke L; lucianekopittke@gmail.com; 0000-0002-6606-7756)

Luiza Marinho Motta Santa Rosa [6] (Rosa, LMMS; luiza.motta26@gmail.com; 0000-0002-4741-4871)

Angelica Gomides dos Reis Gomes, MD, MSc [7] (Gomes, AGR; angelicagrgomes@gmail.com; 0000-0002-4568-0738)

Frederico Bartolazzi, MD, MSc [8] (Bartolazzi, F; fredlazzi@hotmail.com; 0000-0002-9696-4685)

Saionara Cristina Francisco, BSc, MSc [9] (Francisco, SC; saionaracf@gmail.com; 0000-0002-9655-6294)

Felicio Roberto da Costa, MD [2] (Costa, FR; felicio_roberto@hotmail.com; 0000-0001-9923-236X)

Alzira de Oliveira Jorge, MD, MSc, PhD [10] (Jorge, AO; alzira.o.jorge@gmail.com; 0000-0003-1366-1732)

Christiane Corrêa Rodrigues Cimini, MD, MSc, PhD [11] (Cimini, CCR; christiane.cimini@gmail.com; 0000-0002-1973-1343)

Marcelo Carneiro, MD, MSc, PhD [12] (Carneiro, M; marceloc@unisc.br; 0000-0003-3603-1987)

Karen Brasil Ruschel, RN, MSc, PhD [13,14] (Ruschel, KB; karenbruschel@gmail.com; 0000-0002-0812-920X)

Alexandre Vargas Schwarzbold, MD, MSc, PhD [15] (Schwarzbold, AV; alexvspoa@gmail.com; 0000-0002-5535-6288)

Daniela Ponce, MD, MSc, PhD [16] (Ponce, D; daniela.ponce@unesp.br; 0000-0002-6178-6938)

Maria Angélica Pires Ferreira, MD, MSc, PhD [17] (Ferreira, MAPF; mpiferreira@hcpa.edu.br; 0000-0003-0961-524X)

Milton Henriques Guimarães Júnior, MD, MSc [18] (Guimarães-Júnior, MH; miltonhenriques@yahoo.com.br; 0000-0002-2127-8015)

Daniel Vitório Silveira, MD, MSc [19] (Silveira, DV; danielvez@gmail.com; 0000-0002-7381-1651)

Fernando Graça Aranha, MD, MSc [20] (Aranha, FG; fgaranha2012@gmail.com; 0000-0001-9173-8892)

Rafael Lima Rodrigues de Carvalho, BSc, MSc, PhD [21,22] (Carvalho, RLR; rafaelsjdr@hotmail.com; 0000-0003-3576-3748)

Mariana Frizzo de Godoy, MD [23] (Godoy, MF; mfdegodoy@gmail.com; 0000-0002-6631-8826)

Lucas Macedo Pereira Viana[24] (Viana, LMP; lmacedopv@gmail.com; 0000-0002-8457-0531)

Vânia Naomi Hirakata, BSc, MSc[4] (Hirakata, VN; vhirakata@hcpa.edu.br; 0000-0003-4645-2080)

Maria Aparecida Camargos Bicalho, MD, MSc, PhD [25,26,27,28] (Bicalho, MAC; macbicalho@gmail.com; 0000-0001-6298-9377)

Milena Soriano Marcolino MD, MSc, PhD [27,29] (Marcolino MS; milenamarc@gmail.com; 0000-0003-4278-3771)

INSTITUTIONS

[1] Centro Universitário de Belo Horizonte, UniBH. Av. Professor Mário Werneck, 1685, Belo Horizonte, Brazil.

[2] Hospital Metropolitano Odilon Behrens. R. Formiga, 50. Belo Horizonte, Brazil.

[3] Hospital Eduardo de Menezes. R. Dr. Cristiano Rezende, 2213, Belo Horizonte, Brazil.

[4] Hospital Nossa Senhora da Conceição. Av. Francisco Trein, 326, Porto Alegre, Brazil.

[5] Hospital Cristo Redentor. R. Domingos Rubbo, 20, Porto Alegre, Brazil.

[6] Faculdade Ciências Médicas de Minas Gerais. Al. Ezequiel Dias, 275, Belo Horizonte, Brazil.

[7] Rede MaterDei de Saúde. Via Expressa, 15500, Betim, Brazil.

[8] Hospital Santo Antônio. R. Dr. Márcio de Carvalho Lopes, 501, Curvelo, Brazil.

[9] Hospital Metropolitano Dr. Célio de Castro. R. Dona Luzia, 311, Belo Horizonte, Brazil.

[10] Hospital Risoleta Tolentino Neves. R. das Gabirobas, 1, Belo Horizonte, Brazil.

[11] Hospital Santa Rosália. R. Dr. Onofre, 575, Teófilo Otoni, Brazil.

[12] Hospital Santa Cruz. Universidade de Santa Cruz do Sul. R. Fernando Abott, 174, Santa Cruz do Sul, Brazil.

[13] Hospital Universitário Canoas. Av. Farroupilha, 8001, Canoas, Brazil.

[14] Hospital Mãe de Deus. R. José de Alencar, 286, Porto Alegre, Brazil.

[15] Hospital Universitário de Santa Maria. Av. Roraima, 1000, Santa Maria, Brazil.

[16] Hospital das Clínicas da Faculdade de Medicina de Botucatu. Rod. Domingos Sartori, 21500, Botucatu, Brazil.

[17] Hospital de Clínicas de Porto Alegre. R. Ramiro Barcelos, 2350, Porto Alegre, Brazil.

[18] Hospital Márcio Cunha. Av. Eng. Kiyoshi Tsunawaki, 41, Ipatinga, Brazil.

[19] Hospital Unimed-BH. Av. Contorno, 3097, Belo Horizonte, Brazil.

[20] Hospital SOS Cárdio. Rod. SC-401, 121, Florianópolis, Brazil.

[21] Hospital Universitário Professor Edgard Santos. R. Augusto Viana, S/N, Salvador, Brazil.

[22] Escola de Enfermagem da Universidade Federal da Bahia. Basílio da Gama, 241. Salvador, Bahia, Brazil.

[23] Hospital São Lucas da PUCRS. Av. Ipiranga 6690, Porto Alegre, Brazil.

[24] Instituto Nacional de Ciência e Tecnologia Neurotec R. Av. Professor Alfredo Balena, 110, room 114, Belo Horizonte, Brazil.

[25] Hospital João XXIII, Av. Prof. Alfredo Balena, Belo Horizonte, Brazil.

[26] Universidade Federal de Minas Gerais. Av. Professor Alfredo Balena, 110, Belo Horizonte, Brazil.

[27] Fundação Hospitalar do Estado de Minas Gerais, FHEMIG. Al. Vereador Álvaro Celso, 100, Belo Horizonte, Brazil.

[28] Department of Internal Medicine, Medical School & Telehealth Center, University Hospital, Universidade Federal de Minas Gerais. Av. Professor Alfredo Balena, 110, Belo Horizonte, Brazil.

CORRESPONDING AUTHOR

Camila Rabelo Monteiro de Andrade

Centro Universitário de Belo Horizonte, UniBH.

Av. Professor Mário Werneck, 1685, Belo Horizonte, Brazil.

CEP 30455-610

E-mail:camilapep@gmail.co

Supplementary Material 1

Federal University of Minas Gerais

National Multicentre Hospital Registration of Patients with a Disease Caused by SARS-COV-2 (COVID 19)

GUIDANCE MANUAL FOR DATA COLLECTION

Project Coordinated by:
Dr Milena Soriano Marcolino, Federal University of Minas Gerais

Manual Prepared by:
Israel Borges, Karina Prado, Karen Ruschel, Luanna Monteiro, Maíra Viana, Milena Soriano Marcolino, Talita Fischer, Thais Sales

List of Abbreviations

aPPT	Activated Partial Prothrombin Time
ARDS	Acute Respiratory Distress Syndrome
BAV	Atrioventricular Block
BAVT	Total Atrioventricular Block
BMI	Body Mass Index
bpm	Beats per Minute
COPD	Chronic Obstructive Pulmonary Disease
COVID-19	Coronavirus Disease 2019
CRP	C-Reactive Protein
CVA	Cerebral Vascular Accident (Stroke)
DVT	Deep Vein Thrombosis
ECG	Electrocardiogram
ECMO	Extracorporeal Membrane Oxygenation
FiO_2	Fraction of Inspired Oxygen
FM	Face Mask
HCO_3^-	Bicarbonate
HIV	Human Immunodeficiency Virus
IBP	Intra-Arterial Pressure (invasive arterial pressure)
ICU	Intensive Care Unit
IMV	Invasive Mechanical Ventilation
Inc/min	Respiratory Incursions per Minute
INR	International Normalized Ratio
LDH	Lactate Dehydrogenase
LMWH	Low Molecular Weight Heparin
LV	Left Ventricle
MRS	Myocardial Revascularization Surgery
NC	Nasal Catheter
NR	Not Performed
O_2	Oxygen
pCO_2	Partial Carbon Dioxide Pressure
PE	Pulmonary Embolism
AST/GOT	Aspartate Aminotransferase / Glutamic Oxaloacetic Transaminase
ALT/GPT	Alanine Aminotransferase / Glutamic Pyruvic Transaminase
pH	Potential of Hydrogen

pO_2	Partial Oxygen Pressure
QTc	Corrected QT Interval
RT-PCR	Reverse transcription polymerase chain reaction
SAPS-3	Simplified Acute Physiological Score
SARS-COV-2	Severe Acute Respiratory Syndrome - coronavirus 2
SC	Subcutaneous
SDRA	Acute Respiratory Distress Syndrome
SOFA	Sequential Organ Failure Assessment
SV	Supraventricular
TC	Tomography
UFH	Unfractionated Heparin
V-tach	Ventricular Tachycardia

1 Presentation

This manual for completion of the **form** on the electronic platform REDCap® was created to help the applicant, to make this filling-in process easier and quicker. This is a practical tool, in which the main questions on the form are explained in detail, so that the applicant may easily find the instructions and immediately clarify any doubts with regard to the possibilities of responses and the correct way to fill in the form.

The instructions for the answering of the questions follow the **structure** of the form and the order of the questions. In possession of this manual, the applicant can previously become aware of the main questions and refer to the form whenever doubts arise during the application of the questionnaire.

The filling-in of the form, following the instructions in this manual, is essential to allow the identification of different outcomes and make sure of the quality of the evidence that originated from the study.

2 Goal

The goal of this **Guidance Manual** is to present the content of the form of the REDCap® electronic platform for the standardization of the data collection process.

The form, which is the source object of this manual, is the instrument used for the "**National Multicentre Hospital Registration of Patients with a Disease Caused by SARS-COV-2 (COVID 19)**" as approved by CONEP (CAAE 30350820.5.0000.0008).

3 Target Population

The eligible population consists of registrations of patients who have been admitted to hospital institutions (be it through spontaneous demand, transferred from another service, or referred by the pre-hospital service) and who have had a confirmed positive diagnosis for COVID-19 based on detectable RT-PCR or positive IgM in serum testing (conventional serology or rapid test) between March 1 and December 30, 2022.

3.1 Data Collection

The files of eligible patients shall be selected and the data shall be collected in three different moments (admission to hospital, hospitalization period, and hospital discharge or death).

4 Availability and Period of Application

The data shall be collected through an electronic platform (REDCap®). The researchers responsible for data collection shall sign up through the Internet link http://telessaude.hc.ufmg.br/cursos/AcessoRedCap.php.

Once the registration has been made, the coordination of the project shall authorize access by electronic mail (e-mail). The researchers of each institution shall only have access to the data on patients from their own institution. Each researcher must use his or her own password.

The files of eligible patients shall be included on the database as from local ethics approval or when opened by the institution, up to 30 September 2020.

5 Place of Application

The forms shall be filled in at the participating institutions.

6 Responsibility for Filling In the Form

The **form** shall be filled in by the researchers of each institution, as assigned for the project, who have been duly trained in the study protocol and application manual. The data shall be monitored by the coordinator of the registration process. In case of any doubts, the local researchers shall be contacted.

7 Application time

The time needed to fill in the **form** is variable. We estimate that the average time to dill in the form shall be 40 minutes per patient.

8 Questions

The form contains 205 variables, divided into four separate moments of data collection (admittance to hospital, hospitalization, and hospital discharge or death).

9 Methodology

Preparation

The applicants shall access the **form** on the REDCap® electronic platform.

The **form** will be filled in based on data taken from the **medical files** of patients admitted to the partner hospitals (made by reading and extraction of the data present on medical files), the **system of medical tests** (in many institutions, not all tests and tests are copied onto the medical files) and **medical prescriptions**, in a r**etrospective** manner.

Before starting data collection, make sure that you are able to put into practice all the guidance that was supplied during training given by the technical team of the project. Should you have any doubts, please contact the collaborators of the project.

We now present some general guidance about how to proceed while the medical files are being appraised. This guidance is essential to guide the conduct of the researcher while data is being collected:

- Never make any changes to the registration (do not include new information, and do not erase any information that already exists)
- .When data is collected on a physical form, contact the person responsible for the files or the system of medical files at the institution, and book in advance times so that the data may be collected.
- Remember to always carry the consent letter from the institution with you, should there be a need for clarifications with regard to your access to the files. Be prepared to solve any doubts as may occur regarding the execution of the project at the institution.
- For collection of data based on an electronic file, make sure that you still have active access to the system.

Guidance for Data Collection

Form Number

This is an automatic number, generated by REDCap®. If one researcher from a given centre fills in the REDCap® off-line and another researcher fills it in online, then it is possible that the same form numbers may initially be assigned, and then, when the offline data is imported into an online setting, the numbering of the form may be changed.

Centre Number

Each research centre shall have a corresponding 4-digit number (centre number). Make sure of your centre number before you start data collection. In case of any doubt, contact the technical team responsible for the Project.

Identification Number (ID) within the Project

The patient number (ID within the Study) is a field with seven digits, combining the centre number (4 digits) and the sequential identifier of the patient (3 digits). For example: 1001001 is the first patient number corresponding to centre 1001.

Filling in the Form

The form is divided into three different moments of data collection:

1. **Admittance to Hospital:** This includes primary identification and past medical history, clinical appraisal at the moment of admittance to hospital, and additional tests carries out within 24 hours as from the moment of admittance to hospital;
2. **Hospitalization**: This includes clinical evaluation at the moment of admittance to hospital, therapy implemented, support care during the hole hospitalization;
3. **Hospital Discharge or Death**: outcomes.

Type of Response Option

1. **Mandatory:** The variable must be supplied for all patients in all situations (if the information is not available, use "NA").
2. **Non-Mandatory:** The variable is not mandatory, and this can be answered by one or more of the options or by no option of answer. Whenever possible, refrain from leaving an item unanswered, so that the coordination of the Project may be able to distinguish between an absence through forgetting, from unavailable information (use "NA").
3. **Personalized Information**: This is a variable with an open-ended field, to be completed as established in the details within the guidance for completion. Such a variable can be either mandatory or **non-mandatory**.
4. **Conditional:** The variable shall be available depending on the answer to the previous question. For example, on selecting "Sex: Female", the variable "Pregnant" will be accessible, to specify the gravidic condition of the woman. Such a variable can be either mandatory or **non-mandatory**.

It is important to fill in the form supplying as **much information as possible**. On finalizing each part of the form, there is a question about "*form status – complete?*", with the options "*incomplete*", "*unverified*" e "*complete*". After supplying all available data, at the end of the form, you should update the status to "*complete*". Should it not be possible to fill in the whole form at that moment, then you should mark "*incomplete*". Should it be necessary to review the completed form at a later moment, then it is possible to mark it as "*unverified*". This choice shall establish what color shall be assigned to each stage of the form on the "*record home page*", respectively green, red, and yellow.

The variables are detailed as follows:

1. **Admittance to Hospital**

**Primary Identification**		
**Item**	**Options for Answer**	**Guidance for Completion**
ID of the study	Mandatory	Fill in with the **four digits representing the centre number,** followed by the **three digits**, that represent the **patient ID** at the institution, which must be **sequential**

Initials	Mandatory	Supply the **initials of the name and also the initials of the second and last surnames** (for example: for Joseph Anthony Smith, use JAS, for name and surname)
Method of confirmation of COVID-19	Mandatory	Select the options: 1- RT-PCR; 2- Rapid antigen test; 3- Rapid serologic test; 4- Unknown rapid test
Medical File	Mandatory	Supply the number of the medical file or registration at the hospital
Date of Birth	Mandatory	Inform using the DD/MM/YYYY format, or select the date using the calendar.
Sex at Birth	Mandatory	Select one of the options: male or female.
Pregnant	Conditional	Select the option, yes or no. If the ‘male’ option is selected in the previous item, ‘sex at birth’, then this variable shall be inactive.
How many weeks pregnant?	personalized Information	Inform the number of weeks. If ‘no’ was selected in the previous item, or if 'male' was selected as the sex at birth, then this variable shall be inactive.
Date of Admittance to Hospital	Mandatory	Inform the date of admittance to the current hospital institution **If the patient is admitted to hospital for a different reason and then, during the process of hospitalization, starts to show symptoms of COVID-19, which were not present at the moment of admission to hospital, then the date considered as the date of admission to hospital shall be that of onset of symptoms.**
Transferred from another service?	Mandatory	Select the option which best represents the situation of the patient, out of the following: 1 – No; 2 – Emergency Unit; 3 – Hospital institution in the same city; 4 – Hospital institution in a different city; 5 – Campaign Hospital; 6 – No information
City of Residence	Mandatory	Inform the name of the patient’s municipality of origin

**Past History***		
**Item**	**Options for Answer**	**Guidance for Completion**
Vaccination status	Mandatory	Was the patient vaccinated? Select yes or no. If yes, answer above
Which vaccine?	Mandatory	Select the option: 1-Astrazeneca; 2- Coronavac; 3-Janssen; 4-Pfizer; 5- Sputnik; ou 6- Unknown
How many shots?	Mandatory	Check the option: 1- One; 2- Two; 3-Three; 4- Unknown

Date of the last shot	Conditional	Fill in with the date of the last COVID-19 shot DD-MM-AAAA. If only the month available, consider as day 15th (half the month)
Cardiovascular System	Mandatory	Select the option(s) that apply to the patient (multiple answers are accepted): 1- Hypertension, 2- Coronary Artery Disease, 3- Heart Failure, 4- Fibrillation / Atrial flutter; 5- Ischaemic CVA (stroke); 6- Chagas' Disease; 7- Any other cardiovascular diseases (which ones?); 8- No relevant diseases or illness.
Describe any other cardiovascular disease(s) or illness(es)	Conditional	On selecting the 'any other cardiovascular diseases' option, please specify, in this item, the disease or illness shown. If the option 'any other cardiovascular diseases' is not marked, then this item shall not be available.
Respiratory Tract	Mandatory	Select the option(s) that apply to the patient (multiple answers are accepted): 1- Asthma; 2- COPD; 3 – Pulmonary Fibrosis; 4- Active tuberculosis; 5- Past tuberculosis; 6- None of the above.
Metabolic Disorders	Mandatory	Select the option(s) that apply to the patient (multiple answers are accepted): 1 – Diabetes Mellitus; 2 – Obesity (BMI > 30kg/m2); 3 – Neither of the above.
Other Health Conditions	Mandatory	Select the option(s) that apply to the patient (multiple answers are accepted): 1 – Cirrhosis; 2 – Dementia; 3 - Psychiatric Disorders; 4 – Chronic Kidney Disease; 5 – Rheumatologic Disease; 6- Thyroid disturbance; 7 – HIV Infection;86 – Malignant Neoplasm; 9 – Postpartum < 6 weeks; 10 – Prior Transplant; 11 – Other Relevant Health Condition; 12 – No relevant health conditions
Chronic renal failure with need of dialysis?	Conditional	If checked the option "Chronic renal failure", inform if there is any need of dialysis before the COVID-19 hospitalization. Select yes or no.
Which thyroid disease?	Conditional	If checked the option thyroid disease", inform which disease: 1- Hypothyroidism; 2-Hyperthyroidism; 3-Other; 4-Unknown
HIV in treatment?	Conditional	If checked "HIV infection", inform if the patient is in treatment: 1- Yes; 2- No; 3- Unknown
Viral load available? If yes, fill in	Conditional	If checked "HIV infection", fill in the last viral load available in the medical record.
CD4 value available? If yes, fill in	Conditional	If checked "HIV infection", fill in the last CD4 value available in the medical record.
Type of neoplasm	Conditional	If checked "Malignant neoplasm", check the option: 1- Haematologic; 2- Solid organs with metastasis; 3- Solid organs without metastasis or with no information regarding this; 4- No information about type of neoplasm.
Site of the primary neoplasm	Conditional	If checked "Malignant neoplasm", inform the site of the primary neoplasm. If not available, write ND.

Treatment in use	Conditional	If checked "Malignant neoplasm", inform the treatment in use (eg. radiotherapy, chemotherapy and the name of medication)
Type of Transplant	Conditional	If the option "Prior Transplant" has been selected as part of the "Other Health Conditions" item, then please select the option which best represents the type of transplant: 1 – Hematological; 2 – Solid Organs; 3 – No information.
Which organ transplantation?	Conditional	If checked "Solid organ", inform which one.
Describe the other relevant health condition	personalized Information	If you have selected the "Other – which one(s)' option in the "Other Health Conditions" item,. Here please inform the name of the health condition reported, in the event of it being a relevant disease or illness.
Medication of Continuous Use [α]	Mandatory	Select the option(s) applicable to the patient (multiple answers accepted): 1 – Oral anticoagulant; 2- Inhalatory non-steroidal drugs; 3 – Oral non-steroidal drugs; 4 – Immunosuppressants; 5 – Does not use medication
Lifestyle	Mandatory	Select the option(s) applicable to the patient. Multiple answers accepted): 1 – Illicit Drugs; 2 – Alcohol Consumption; 3 – Current smoker; 4 – Former smoker; 5 – None of the above.
Functional status**	Mandatory	Select the option referring to the patient before falling ill with Covid-19: 1- Robust - includes very active (he exercised regularly), active (no active symptoms of the disease, he exercised occasionally) and regular (health issues well controlled); 2- Vulnerable or mild frailty - not dependent, but slower, tired throughout the day; or need help with instrumental activities (finance, transport, work domestic, medicines); 3- Moderately frail (needs help with bathing and support to dress); 4- Severely or very severely frail (totally dependent on ADLs); 5- Terminally ill (life expectancy < 6 months); 6- No information

* For purposes of **past history**, the following definitions shall be considered:

** Functional status was assessed through an adapted version of the Clinical Frailty Scale, a widely employed screening tool. This variable was included in the protocolo in 2021.

(Ref ROCKWOOD, 2005)

Comorbidities

- **Cardiovascular System:**

- **Hypertension**: prior diagnosis of systemic hypertension as reported on a medical file, and/or use of medication against hypertension, regardless of whether this is regular or irregular treatment, and on whether the blood pressure is controlled or not.
- **Arterial Coronary Disease**: History of prior angioplasty, myocardial revascularization surgery (MRS), acute heart attack, or angina.
- **Heart Failure**: Annotations on medical records confirming heart failure, regardless of whether the ejection fraction is preserved or has been reduced.
- **Atrial Fibrillation or Atrial Flutter**: Annotation of any atrial fibrillation (paroxystic or permanent) or atrial flutter, in the medical records.
- **Ischaemic CVA**: Past history of cerebral ischaemia, noted down in medical records

- **Respiratory Tract:**
  - **Asthma**: Reports of asthma or "bronchitis", with compatible prior symptoms, in adulthood, regardless of the use of medication for control (such as corticoids/LABA), in the case of adults. For pediatric patients, it should be considered as asthma whenever there has been an asthmatic crisis or a bout of "bronchitis", at any moment within childhood.
  - **CPOD**: Report of COPD regardless of the use of inhalatory medication for control, but with compatible risk factors (Example: cigarette smoking > 20 years/pack, or prolonged use of wood ovens) OR patients using inhalatory medicines compatible with the treatment of COPD (LABA, LAMA, or a combination of these, whether associated to inhalatory corticoids or not) and associated risk factors.
  - **Pulmonary Fibrosis:** Registered on medical file.
  - **Active tuberculosis:** Registered on medical file
  - **Past tuberculosis:** Registered on medical file

- **Metabolic Disorders:**
  - **Diabetes Mellitus**: Diabetes reported on the medical file, of whatever type, be it insulin-dependent or not.
  - **Obesity**: BMI > 30Kg/m^2 registered on medical file or as past medical history

- **Medication for Continuous Use:** Mark the medication that the patient is taking, according to the medical records.

- **Lifestyle – Medical File Information**:
  - Alcohol Consumption: When reported on the medical file, except when there is a report mentioning 'social drinking' or a consumption of up to two standards units of drink per day: 2 cans of beer, 2 doses of Brazilian firewater (*cachaça*), two glasses of wine, or two glasses of whisky.
  - Present and past smoker: Mentioned on medical file, regardless of time and quantity;
  - Use of other illicit drugs: Reported in medical file, regardless of quantity.

[a]With regard to medication, the following list presents the most significant examples within each class:

Class	Medication
Oral Anticoagulants	Apixaban, Dabigatran, Edoxaban, Rivaroxaban, Warfarin.
Oral Corticoids	Betamethasone, Dexamethasone, Prednisolone, Prednisone, Deflazacort.
Inhalatory Corticoids	Beclomethasone, Budesonide, Ciclesonide, Dexamethasone, Fluticasone, Mometasone, Triancinolone.
Imunossupressants	Azatioprin, Cyclophosphamide, Cyclosporin, Everolimos, Methotrexate, Mycophenolate Sodium, Mycophenolate Mophetil, Sirolimos, Tacrolimos.

2. Clinical assessment on ICU admission

Clinical Assessment on Admission		
**Item**	**Options for Answer**	**Guidance for Completion**
Date of Onset of First Symptom	Mandatory	Inform the date of onset of first symptom using the DD/MM/YYYY format, or fill in the date using the calendar.
Main reason of admission	Mandatory	Check one<br>1- Covid-19; 2- Other reason
What was the other reason for hospitalization?	Conditional	If checked “other reason”, inform which one(s): 1- Cardiogenic shock; 2- Coronary artery disease; 3- Cerebrovascular disease; 4- Hepatic encephalopathy; 5- Gastrointestinal hemorrhage; 6- Decompensated heart failure; 7- Infection; 8- Sepsis; 9- Septic shock; 10- Trauma; 11- Pulmonary embolism; 12- Labor; 13- Other
Which other?	Conditional	If checked other above, write which one
Was the patient suspicious of Covid-19 when admitted?	Mandatory	If hospitalization for other reasons, answer yes or no.
Clinical signs and symptoms	Mandatory	Please select the options that apply to the patient (multiple responses are accepted): 1 – Adinamia; 2 – Ageusia (loss of taste); 3 – Anosmia (loss of smell); 4 – Arthralgia; 5 – Headache; 6 – Coryza; 7 – Diarrhea; 8 – Dyspnoea; 9 – Sore throat; 10 – Fever; 11 – Haemoptysis; 12 – Hyporexia; 13 – Neurological symptoms; 14 – Myalgia; 15 – Nausea and vomiting; 16 – Cough; 17 - No Symptoms; 18 – Others
What neurological symptoms?	Conditional	If checked “neurological symptoms”, check which one (ones): 1- Stroke; 2- Seizure; 3- Delirium; 4-Others

Which other neurological symptoms?	Conditional	If checked “others” above, describe which ones.
Which other signs or symptoms?	Conditional	If you have checked “others” in “clinical signs and symptoms”, please describe which ones.
Was the patient admitted straight to the ICU, without passing through the emergency department or other place?	Mandatory	Fill in the yes or no option. In this case, you must mark the option yes only if there is no clinical examination before the ICU. If yes, the next variables related to the clinical evaluation on admission and laboratory findings on admission are inactive, and the next variable to be filled will be in the form related to ICU admission
Glasgow Coma Scale	Mandatory / personalized Information	Please fill in with the patient’s Glasgow score at the moment of admission. If not available, then please use ND. **If there is a record on the file of an alert and lucid patient, or use of the abbreviations LOTE, BOTE or LOCV, then consider a Glasgow score of 15.** **If the patient is under continuous sedation, then fill in with NA (not applicable).**
State / Level of Consciousness	Conditional	If the information about the Glasgow Coma Scale is not complete or not available, then please select the option(s) that best apply to the patient, choosing between: 1 – Lucid and alert; 2 – Confused; 3 -Disoriented; 4 – Drowsy; 5 – In a state of torpor; 6 – Comatose. **If the patient is in a state of continuous sedation, then please mark “comatose”.**
Systolic Blood Pressure	Mandatory / personalized Information	Inform the systolic blood pressure in mmHg. Complete with NA if not available.
Diastolic Blood Pressure	personalized Information	Inform the diastolic blood pressure in mmHg. Complete with NA if not available.
If there is monitoring of IBP; mean arterial pressure (mmHg)	personalized Information	If this is a patient with monitoring of invasive arterial pressure, then please inform the mean arterial blood pressure. Put NA if the information is not available.
Is there any use of vasoactive amines?	Mandatory	Fill in with either ‘yes’ or ‘no’ at the moment of confirmation of the pressure as registered in the previous items.
Heartbeat Frequency	Mandatory/ personalized Information	Supply the value of the heartbeat frequency in beats per minute (bpm). Put NA if the information is not available.
Respiratory rate	Mandatory/ personalized Information	Supply the breathing frequency in Inc/min. Put NA if the information is not available.
Temperature	personalized Information	Inform the temperature in ºC. Put NA if the information is not available.
Saturation of O_2	Mandatory/ personalized Information	Inform the level of saturation of O_2 in %. Put NA if the information is not available.
Environmental Air	Mandatory	Select one of the options, ‘yes’ or ‘no’, according to the supply of O_2 when the saturation of the previous item is registered.
Nasal Catheter (NC)	Mandatory/ Conditional	If the ‘no’ option is selected in the ‘Environmental Air’ item, then the option of oxygen supply through a NC shall become available. Select one of the options, ‘yes’ or ‘no’
Flow	personalized Information	Inform the flow (L/min) of O_2 offered by the nasal catheter. This item shall only be available for completion if ‘yes’ is selected in the previous item. Put NA if the information is not available.

Face Mask	Mandatory/ Conditional	If the 'no' option is selected in the 'Environmental Air' and 'Nasal Catheter' items, then the option of supply of oxygen through FM shall become available. Select one of the options: 'yes' or 'no'.
Flow	personalized Information	Inform the flow (L/min) of O_2 offered by the face mask. This item shall only be available if 'yes' is selected in the previous item. Put NA if the information is not available.
Invasive mechanical ventilation	Mandatory/ Conditional	If the 'no' option is selected in the 'Environmental Air', 'Nasal Catheter', and 'Face Mask' items, then the option of supply of oxygen through IMV shall become available. Select one of the options: 'yes' or 'no'.
Which FiO_2?	Conditional	If in IMV, fill in the FiO_2 registered in the medical record.

**Laboratory Findings on ICU Admission**		
**Item**	**Options for Answer**	**Guidance for Completion**
Hemoglobin (g/dL)	personalized Information	Inform the value of hemoglobin content on the CBC made at the admission of the patient to hospital. Put NA if this laboratory test is not available.
Leukocytes (cells/mm^3)	Mandatory/ personalized Information	Inform the absolute global leukocyte count on the CBC made at the admission of the patient to hospital. Put NA if this laboratory test is not available.
Neutrophils (cells/mm^3)	personalized Information	Inform the value of hemoglobin content on the CBC made at the admission of the patient to hospital. Put NA if this laboratory test is not available.
Lymphocytes (cells/mm^3)	Mandatory/ personalized Information	Inform the absolute lymphocyte count as on the CBC made at the admission of the patient to hospital. Put NA if this laboratory test is not available.
Platelets (cells/mm^3)	Mandatory/ personalized Information	Inform the absolute platelet count as in the test made at the admission of the patient to hospital. Put NA if this laboratory test is not available.
Total Bilirubin (mg/dL)	Mandatory/ personalized Information	Inform the total bilirubin value as in the test made at the admission of the patient to hospital. Put NA if this laboratory test is not available.
Creatinine (mg/dL)	Mandatory/ personalized Information	Inform the creatinine level as in the test made at the admission of the patient to hospital. Put NA if this laboratory test is not available.
D-dimer (ng/mL)	Mandatory/ personalized Information	Inform the D-dimer value as in the test made at the admission of the patient to hospital. Put NA if this laboratory test is not available.
D-dimer reference value	Mandatory/ personalized Information	Inform the D-dimer maximum reference value.
Ferritin (ng/mL)	Mandatory/ personalized Information	Inform the ferritin value as in the test made at the admission of the patient to hospital. Put NA if this laboratory test is not available.
Lactate	Mandatory	Fill in this field if the lactate is: 1 – Arterial; 2 – Venous; 3 – Not applicable, if the test is not performed.

Lactate Unit	personalized Information	Inform the measuring unit as registered in the previous unit (mmol/L or mg/dL) used as part of the tests performed on the admission of the patient to hospital.
CRP (mg/L)	personalized Information	Inform the CRP value in the test made on the patient's admission to hospital. Put NA in the test is not available.
aPPT (seconds) / control	personalized Information	Inform the aPTT value in the test made on the patient's admission to hospital. Put NA if the test is not available.
RNI	personalized Information	Inform the RNI value in the test made on the patient's admission to hospital. Put NA if the test is not available.
Sodium (mmol)	Mandatory/ personalized Information	Inform the Sodium level in the test made on the patient's admission to hospital. Put NA if the test is not available.
GOT/AST (U/L)	Mandatory/ personalized Information	Inform the GOT/AST level in the test made on the patient's admission to hospital. Put NA if the test is not available.
GPT/ALT (U/L)	Mandatory/ personalized Information	Inform the GOT/ALT level in the test made on the patient's admission to hospital. Put NA if the test is not available.
Troponin	personalized Information	Inform the troponin level in the test made on the patient's admission to hospital. Put NA if the test is not available.
Troponin reference value	personalized Information	Inform the maximum troponin reference value.
Urea (mg/dL)	personalized Information	Inform the urea level in the test made on the patient's admission to hospital. Put NA if the test is not available.
pH	personalized Information	Inform the pH as part of the arterial gasometry test, on the patient's admission to hospital. Put NA if the test is not available.
Arterial pCO_2	personalized Information	Inform the pCO_2 value within the arterial gasometry test on the patient's admission to hospital. Put ND if the test is not available.
Arterial pO_2	Mandatory/ personalized Information	Inform the pCO_2 value within the arterial gasometry test on the patient's admission to hospital. Put ND if the test is not available.
HCO_3^-	personalized Information	Inform the HCO_3^- value within the arterial gasometry test on the patient's admission to hospital. Put ND if the test is not available.
FiO_2 (at the moment of collection of gasometrics)*	Mandatory/ personalized Information	Inform the FiO_2 value at the moment of collection for the arterial gasometry test on the patient's admittance to hospital. Put NA if the test is not available. **This item of data is essential for the calculation of the ratio between PaO2/FiO2, meaning that it is very important to try to obtain this.**

* If the patient is not in mechanical ventilation, the use of the following estimates for FiO_2 has been agreed:

Device	Flow (L/min)	Approximate value of FiO2 to be used in the collection form
**No – regular room air**	0	0.21
**Nasal Cannula**	1	0.24
	2	0.28
	3	0.32
	4	0.36
	5	0.40
	6	0.44
**Simple Mask**	5	0.40
	6	0.50
	7	0.60

**Mask with Non-Reinhaling Reservoir**	6	0.60
	7	0.70
	8-9	0.80
	10-15	0.95

3. hospitalization

Therapeutic Intervention		
**Item**	**Options for Answer**	**Guidance for Completion**
Therapies used during hospitalization	Mandatory	Select the option(s) referring to the therapy used during the whole period of hospitalization of the patient (multiple answers are accepted):\ 1 – Antibiotics for nosocomial infections; 2 – Anticoagulants; 3 – Antifungal Drugs; 4 – Neuromuscular blocker for intubation; 5 – Oral or IV corticotherapy; 6 – Inhaled corticoids; 7 – None of the Above.
Which Anticoagulant?	Mandatory/ Conditional	If the ‘Anticoagulant’ option has been chosen in item 128, then select the item that refers to what anticoagulant was used: 1 – Unfractionated Heparin; 2 – Low Molecular Weight Heparin; 3 – Fondaparinux; 4 – Warfarin; 5 – Others.
Unfractionated Heparin Dose*	Mandatory/ Conditional	If the option ‘Unfractionated Heparin’ is selected in item 128.1, please select the option referring to the dose of anticoagulants: 1 – Prophylactic; 2 – Therapeutic
Start of Treatment with Unfractionated Heparin	Mandatory/ personalized Information	If the option ‘Unfractionated Heparin’ is selected in item 128.1, please inform the date when the medication started to be used, in DD/MM/YYYY format.
End of Treatment with Unfractionated Heparin	Mandatory/ personalized Information	If the option ‘Unfractionated Heparin’ is selected in item 128.1, please inform the date when the medication stopped being used, in DD/MM/YYYY format.
Dose of Low Molecular Weight Heparin[α]	Mandatory/ Conditional	If the option ‘Low Molecular Weight Heparin’ is selected in item 128.1, please select the option referring to the dose of anticoagulants: 1 – Prophylactic; 2 – Therapeutic
Start of Treatment with Low Molecular Weight Heparin	Mandatory/ personalized Information	If the option ‘Low Molecular Weight Heparin’ is selected in item 128.1, please inform the date when the medication started to be used, in DD/MM/YYYY format.
End of Treatment with Low Molecular Weight Heparin	Mandatory/ personalized Information	If the option ‘Low Molecular Weight Heparin’ is selected in item 128.1, please inform the date when the medication stopped being used, in DD/MM/YYYY format.
Dose of Fondaparinux	Mandatory/ Conditional	If the option ‘Fondparinux’ is selected in item 128.1, please select the option referring to the dose of anticoagulants: 1 – Prophylactic; 2 – Therapeutic
Start of Treatment with Fondparinux	Mandatory/ personalized Information	If the option ‘Fondparinux’ is selected in item 128.1, please inform the date when the medication started to be used, in DD/MM/YYYY format.
End of Treatment with Fondparinux	Mandatory/ personalized Information	If the option ‘Fondparinux’ is selected in item 128.1, please inform the date when the medication stopped being used, in DD/MM/YYYY format.

Start of Treatment with Warfarin	Mandatory/ personalized Information	If the option 'Warfarin' is selected in item 128.1, please inform the date when the medication started to be used, in DD/MM/YYYY format.
End of Treatment with Warfarin	Mandatory/ personalized Information	If the option 'Warfarin' is selected in item 128.1, please inform the date when the medication stopped being used, in DD/MM/YYYY format.
What other anticoagulant?	Conditional/ personalized Information	If the option 'Others' is selected in item 128.1, please inform which other anticoagulant is being used.
Start of Treatment with Other Anticoagulant	Mandatory/ personalized Information	If the option 'Other Anticoagulant' is selected in item 128.1, please inform the date when the medication started to be used, in DD/MM/YYYY format.
End of Treatment with Other Anticoagulant	Mandatory/ personalized Information	If the option 'Other Anticoagulant' is selected in item 128.1, please inform the date when the medication stopped being used, in DD/MM/YYYY format.
How is the neuromuscular blocker used?	Mandatory/ personalized Information	If selected the option "neuromuscular blocker", choose one: 1- Bolus; 2- Continuous infusion; 3- No information
For how many days was the neuromuscular blocker used?	Mandatory/ personalized Information	If selected the option "neuromuscular blocker", fill in for how many days.
Which Corticoid?	Mandatory/ Conditional	If the option 'Corticoid' is selected in item 128, then please mark if the corticoid used was: 1 – Dexamethasone, 2 – Another Corticoid.
Start of Treatment with Corticoid	Conditional/ personalized Information	If the option 'Corticoid' is selected in item 128, please inform the date when the medication started to be used, in DD/MM/YYYY format.
Duration of the Treatment with Corticoid (days)	Conditional/ personalized Information	If you select the option "corticoid", fill in how many days. If the patient used more than one type of corticoid, add the total use days. The day of start is day 1
Dose of Dexamethasone (mg/dia)?	Mandatory/ personalized Information	If you select the option "dexamethasone", put the diary dose.
Dose of hydrocortisone (mg/dia)?	Mandatory/ personalized Information	If you select the option "hydrocortisone", put the diary dose.
Dose of methylprednisolone (mg/dia)?	Mandatory/ personalized Information	If you select the option "methylprednisolone", put the diary dose.
Dose of prednisone or prednisolone (mg/dia)?	Mandatory/ personalized Information	If you select the option "prednisone or prednisolone", put the dairy dose.
Dose of other corticosteroid (mg/dia)?	Mandatory/ personalized Information	If you select the option "other corticosteroid", put the diary dose.
Therapy Introduced Specifically for Covid-19	Mandatory	Select the option(s) referring to the therapies as used during the whole of the hospitalization period (multiple answers are accepted): 1 – Antibiotic in the acute phase; 2 – Immunoglobulin; 3 – Convalescent Plasma; 4 – Remdesivir; 5 – Sarilumab; 6 – Tocilizumab; 7 – Other (Which?) 8 – None.
Describe Other Therapy Used	Mandatory/ personalized Information	If the option 'Other – which one?' has been selected under 'Therapy Introduced Specifically for COVID-19", please describe the other therapy used.
Support Care	Mandatory	Select the option(s) related to support care as provided during the whole period of hospitalization: 1 – Vasoactive

		Amines; 2 – ECMO; 3 – Respiratory physiotherapy; 4 - Motor physiotherapy 5 - Prone position (non-IMV); 6- Prone position (on IMV); 7 – Volemic Resuscitation; 8 – Non-Invasive Mechanical Ventilation; 9 – None of these
Date and time of the start of ECMO	Conditional	If checked “ECMO”, inform the date and time of the start of ECMO.
Date and time of the indication of ECMO	Conditional	If checked “ECMO”, inform the date and time of the indication of ECMO.
Date of the end of ECMO	Conditional	If checked “ECMO”, inform the date of the end of ECMO.
Were there any ECMO complications?	Conditional	If checked “ECMO”, answer with yes or no..
Which ECMO complications?	Conditional	Se assinalado “sim” na variável anterior, descrever qual complicação
Definition of Palliative Care for the Patient	Mandatory	Answer with ‘yes’ or ‘no’.
Was the definition of Palliative Care for the Patient established at the moment of admission?	Mandatory	If checked ”yes” above, inform if the indication was clearly established at hospital admission: 1- Yes; 2- No; 3- Not clear

* **Dose of unfractionated heparin (UFH):** Doses considered **prophylactic** are UFH 5,000 UI applied subcutaneously (SC) every 12 hours (12/12h) or every 8 hours (8/8h). The **therapeutic** dose is normally given based on the weight of the patient, in a continuous infusion pump, with monitoring of aPPT. In exceptional cases, there are therapeutic doses at 320UI/kg of attack weight and 250UI/kg of weight 12/12h SC, without any monitoring of aPPT.

Dose of low molecular weight heparin: Examples of **prophylactic** doses: enoxaparin 40mg SC 24/24h or dalteparin 5000UI 24/24h. Examples of **therapeutic** doses include: enoxaparin 1mg/Kg of body weight SC 12/12h or 1.5mg/Kg of body weight, every 24 hours; dalteparin 200UI/Kg SC every 24 hours. The therapeutic dose could be adjusted if there is kidney failure.

Dose of fondaparinux: The prophylactic dose of fondaparinux is 2.5 mg SC every 24 hours. The therapeutic dose depends on weight, being set at 5mg if <50Kg, 7.5mg if 50-100Kg, and 10mg if >100Kg, SC, every 24 hours.

1. **Discharge or Death**

Outcomes		
**Item**	**Options for Answer**	**Guidance for Completion**

Date of Hospital Discharge / Death / Transfer	Mandatory	Inform the date of hospital discharge or death, using the DD/MM/YYYY format.
Was there a transfer to another institution?	Mandatory	Answer 'yes' or 'no', with regard to transfer to another institution.
Which Institution?	Mandatory/ Conditional	If the answer to the previous item was 'yes', then describe the institution of transfer.
Was there any need for mechanical ventilation?	Mandatory	Answer 'yes' or 'no', with regard to the need for mechanical ventilation
Date of intubation	Conditional	If checked for IMV, fill in with the date of the first intubation.
Days of mechanical ventilation	Mandatory/ personalized Information	If the answer to the previous item is 'yes', then please inform the number of days when the patient remained under mechanical ventilation.
Was there any need for a tracheostomy?	Mandatory/ personalized Information	Answer 'yes' or 'no'.
Was there any need for replacement kidney therapy (dialysis)?	Mandatory	Answer 'yes' or 'no', with regard to the need for dialysis during the hospitalization period.
Intercurrences during hospitalization?	Mandatory	Select the option(s) that correspond to the support care provided during the whole hospitalization period (multiple answers are accepted): 1. Arrhythmia; 2- Pneumonia with bronchiolitis (check CT thorax); 3- Septic Shock; 4 - Acute HF (new case, or decompensated chronic case); 5 - Hospital Infection; 6 - Heart Attack; 7 - Renal failure; 8 - Myocarditis; 9 - Pericarditis; 10 - Haemorrhage; 11 - ARDS; 12 - Vascular Thrombosis; 13 - Other; 14 - None
Which arrhythmia?	Mandatory/ Conditional	If you checked "1- Arrhythmia" check which ones:: 1- Fibrilação/ atrial flutter; 2- atrioventricular block with need of pacemaker; 3- Multifocal atrial rhythm; 4- Supraventricular tachycardia; 5- Monomorphic ventricular tachycardia; 6- Polymorphic ventricular tachycardia; 7-Other.
Site of the Haemorrhage?	Mandatory/ Conditional	If 'Hemorrhage' was selected as part of 'Intercurrences during hospitalization', then please inform the site of the hemorrhage.
How serious was the hemorrhage?	Mandatory/ Conditional	If 'Hemorrhage' was selected as part of 'Intercurrences during hospitalization', then choose one of the following alternatives: 1. Serious; 2. Not serious, but clinically relevant; 3. Not serious.
What kind of thromboembolic event?	Mandatory/ Conditional	If 'Vascular Thrombosis' was selected under 'Intercurrences During hospitalization', then select one option corresponding to the type of thromboembolic event: 1 – DVT; 2 – TEP; 3 – Arterial Thrombosis.
Any other complications?	Mandatory/ Conditional	If 'Other' was selected under 'Intercurrences During hospitalization', then please select the other complication that was shown.
Any gestational complications?	Mandatory/ Conditional	If pregnant, answer yes or no.
Which gestational complication?	Mandatory/ Conditional	Select which complications: 1- Miscarriage; 2- Ectopic gestation; 3- Pre-eclampsia; 4- Eclampsia; 5-HELLP syndrome; 6-

		Puerperium or labor hemorrhage;7- Hysterectomy, 8- Puerperal infection; 9-Other
Which other gestational complication?	Mandatory/ Conditional	If you checked “other” above, inform which one.
labor during hospitalization?	Mandatory/ Conditional	Answer yes or no.
Type of labor	Mandatory/ Conditional	If checked yes above, check the type of labor:1- Natural childbirth; 2- Cesarean delivery; 3- Unknown
Was the child born alive?	Mandatory/ Conditional	Answer yes or no.
Weight of the newborn (g)	personalized Information	Fill in the weight of the newborn.
APGAR on the 1st minute	personalized Information	Fill in the APGAR score on the 1st minute
APGAR on the 5th minute	personalized Information	Fill in the APGAR score on the 5th minute
Death	Mandatory	Answer with ‘yes’ or ‘no’.

* **Need for mechanical ventilation:** Annotation on the medical record, confirming the need for mechanical ventilation

[α] **Fault of extubation:** Record on the medical file, confirming fault of extubation.

[ƚ] **Need for replacement kidney therapy:** An annotation on the medical records mentioning dialysis during hospitalization that was started during hospitalization (meaning that prior dialytics are excluded).

[Ŧ] Consider the following definition for intercurrences during hospitalization:

1. **Septic Shock:** Annotation on the medical file mentioning septic shock; or, in the case of patients with evidence of infectious process: presence of shock; use of amines and lactate persistently over 2 mmol/L (18 mg/dL), despite appropriate volemic resuscitation
2. **Widespread Intravascular Coagulation:** Annotation on medical files or score as proposed by the *International Society on Thrombosis and Haemostasis* of ≥5, with this score being calculated automatically based on the information supplied on the form, taking into account factors such as platelet count, D-dimer, coagulogram and fibrinogen.
3. **Acute heart failure:** A new, or a decompensated chronic case of heart failure, regardless of whether the ejection fraction is preserved or reduced.
4. **Hospital Infection:** Record of a bacterial infectious process in any site, or at an undetermined site, diagnosed 48 hours after admission to hospital.
5. **Heart Attack (Myocardial Infarction)**: Medical files recording an acute myocardial infarction of any type.
6. **Renal failure:** diagnosis of acute kidney injury (or failure) recorded in medical records, or an increase of at least 0.3 mg/dL in creatinine, in relation to baseline creatinine.
7. **Myocarditis:** Diagnosis of myocarditis, duly recorded on the medical file.
8. **Pericarditis:** Diagnosis of pericarditis, duly recorded on the medical file.

9. **Hemorrhage:** Medical file mentioning hemorrhagic complication(s) which could be considered as (Variable 146.2):

- **Serious Hemorrhage:** Clinically evident bleeding leading to any of the following situations: death; involvement of a key anatomic site (intracranial; spinal; pericardial; articular; retroperitoneal; or intramuscular, with compartment syndrome); fall of at least 2 g/dL in the concentration of hemoglobin; shock; transfusion of at least 2 units of entire blood or RBC concentrate; or permanent invalidity.
- **Hemorrhage not serious, but clinically relevant:** Presence of evident bleeding that does not meet the criteria for defining serious bleeding, but which warranted medical intervention, temporary interruption of treatment, or that generated any kind of pain.
- **Hemorrhage not serious:** Do not fall under any of the previous criteria.

10. **Adult Respiratory Distress Syndrome:** Medical file mentioning adult breathing distress syndrome, ARDS, SDRA; or a diagnosis of disproportional hypoxaemia, registered on the medical file, through arterial gasometry with $pO_2/FiO_2<200$ at any moment; or through maneuvers of alveolar recruitment.

11. **Vascular Thrombosis:** Diagnosis of arterial thrombosis registered on a medical file; deep venous thrombosis confirmed by imaging test (duplex scan or compression ultrasound); and/or pulmonary embolus by imaging test (angiotomography, scintillography; or, if there is haemodynamic instability and in the absence of confirmation through previous testing, changes that would suggest acute overload of the right ventricle, in an echocardiogram or bedside ultrasound).

12. Gestational complications:

1- Miscarriage: termination of pregnancy before the 20th week of gestational age.

2- Ectopic pregnancy: select this option if there is a description in the pregnancy record that develops outside the uterine cavity.

3- Pre-eclampsia: description of pre-eclampsia in the medical record.

4- Eclampsia: description in the eclampsia chart.

5- HELLP syndrome: description in the medical record of HELLP syndrome (hemolysis, increased liver enzymes and thrombocytopenia.

6- Hemorrhage in childbirth or puerperium: excessive loss of blood after childbirth, which has been described in the medical record.

7- Hysterectomy: postpartum uterus removal procedure due to complications of childbirth, as described in the chart.

8- Puerperal infection: any infection of the genital tract that occurs during the puerperium. Examples: endometritis, episiotomy infection (EPIS) or vaginal lacerations, surgical site infection.
